# Extracting disorder parameters from optical spectra of non-fullerene acceptors†

**DOI:** 10.1039/d5mh00547g

**Published:** 2025-07-07

**Authors:** Siebe Frederix, Samuele Giannini, Melissa Van Landeghem, David Beljonne, Koen Vandewal

**Affiliations:** a Institute for Materials Research (imo-imomec), Hasselt University Martelarenlaan 42 B-3500 Hasselt Belgium siebe.frederix@uhasselt.be koen.vandewal@uhasselt.be; b Imec, imo-imomec Wetenschapspark 1 B-3590 Diepenbeek Belgium; c Energyville, imo-imomec Thor Park 8320 B-3600 Genk Belgium; d Institute of Chemistry of OrganoMetallic Compounds, National Research Council (ICCOM-CNR) via G. Moruzzi 1 I-56124 Pisa Italy; e Laboratory for Chemistry of Novel Materials, University of Mons Place du Parc 20 B-7000 Mons Belgium

## Abstract

Organic solar cells have seen significant advancements through the use of non-fullerene acceptors, yet understanding the impact of molecular design on energetic disorder remains critical for optimizing material performance. In this work, we investigate three methodologies for quantifying static and dynamic excitonic disorder by analysing the temperature dependence of spectral features in thin film absorption and photoluminescence spectra. Our results demonstrate that fitting the temperature dependence of the first emission peak energy is the most reliable approach for assessing static disorder, while linewidth fitting of absorption spectra is best suited for quantifying dynamic disorder. Comparative case studies reveal that linear *n*-octyl side chains (*e.g.*, in O-IDTBR and IDIC-4Cl) improve aggregation and induce the lowest static disorder, whereas bulkier side chains (*e.g.*, 2-ethylhexyl and phenylhexyl) result in static disorder parameters which are approximately 50% larger in magnitude. For materials exhibiting strong aggregation, such as Y6, the limitations of current models underscore the need for caution when interpreting disorder metrics. These findings highlight the importance of side chain engineering in controlling the excitonic energetic landscape and provide guidance for the accurate assessment of the related disorder parameters in organic semiconductors.

## Abstract 2

Excitonic disorder in NFA thin films is investigated through temperature-dependent optical spectroscopy, linking molecular design to disorder parameters.

## Graphical abstract

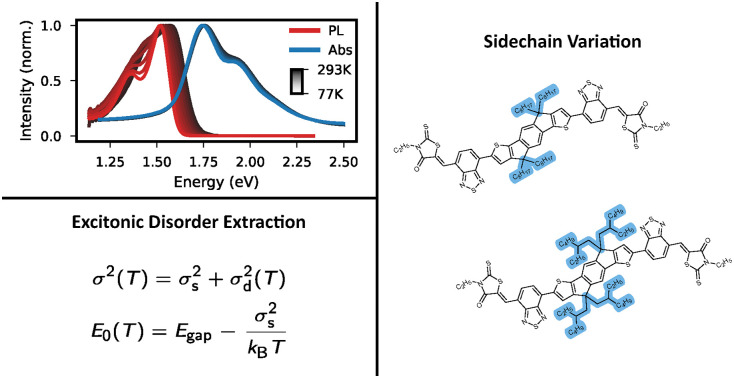

**New concepts**

Understanding and controlling energetic disorder is essential for optimizing the performance of organic solar cells (OSCs). In this study, we focus on thin films of non-fullerene acceptors where we systematically evaluate three methodologies for extracting static and dynamic excitonic disorder from the temperature dependence of optical spectra. Our findings reveal that analysis of the peak energy shifts in photoluminescence provides the most reliable way for obtaining static disorder, whereas linewidth analysis in absorption spectra is best suited for dynamic disorder quantification. Additionally, we demonstrate how molecular design, particularly side chain engineering, influences disorder parameters, with linear *n*-octyl side chains reducing disorder and bulkier groups increasing it. Finally, we highlight fundamental limitations of these methodologies in strongly aggregating materials, such as Y6, where intermolecular interactions alter spectral features. This study provides a refined framework for experimentally assessing energetic disorder in non-fullerene acceptors, offering practical guidance for future materials design and characterization.

## 1 Introduction

Organic solar cells (OSCs) have emerged as a promising technology for renewable energy, offering advantages such as flexibility, lightweight construction, and tuneable properties.^1,2^ Recent advancements in OSCs have been driven by the development of non-fullerene acceptors (NFAs), which have enabled higher efficiencies and greater versatility in molecular design compared to traditional fullerene-based materials.^3^ Moreover, state-of-the-art devices have now reached the milestone of 20% power conversion efficiency.^4^ However, the performance of OSCs is intrinsically linked to energetic disorder.^5^ The energetic disorder in OSCs has two main origins: disorder which can be attributed to the donor and/or acceptor, and disorder related to the donor–acceptor complex, usually referred to as charge-transfer (CT) disorder. Both types of energetic disorder are known to influence OSC performance.^5–7^ The disorder of the donor and acceptor phases is expected to mainly influence electron and hole mobilities, which in turn negatively impacts the fill factor of OSCs. CT disorder, on the other hand, will affect photophysical processes at the donor–acceptor interface such as geminate and non-geminate recombination which affect the fill factor and non-radiative voltage losses.^8^ Understanding and quantifying energetic disorder is therefore crucial for optimizing OSC performance and elucidating the relationship between molecular structure and device functionality.

Energetic disorder in organic semiconductors is commonly quantified using two distinct figures of merit: the Urbach energy and the static energetic disorder derived from the Gaussian Disorder Model (GDM).^9–11^ The Urbach approach has its origins in inorganics where Franz Urbach made a first report of exponential absorption tails in AgBr crystals.^12^ In the Urbach approach, it is assumed that absorption below the gap of the system follows an exponential decay with decreasing photon energy. The steepness of this exponential tail, characterized by the Urbach energy *E*_U_, reflects the degree of disorder—broader tails correspond to more disordered systems. On the other hand, the GDM assumes that the excitonic or CT density of states (DOS) is normally distributed, where the standard deviation *σ*_s_ serves as a measure of static energetic disorder. The subscript “s” denotes the static nature of the disorder, attributed to conformational and environmental variation of the molecules in the solid. The Urbach energy serves as a general disorder parameter of the whole system, whereas *σ*_s_ can make a distinction between the nature of the states (*i.e.* excitonic or CT). Both models can be extended to account for dynamic disorder, which originates from thermally activated low-frequency molecular motions and contributes to the overall energetic landscape at finite temperatures.^13,14^

Over the years, various experimental techniques have been developed to extract these disorder parameters. Urbach energies are often obtained from photovoltaic external quantum efficiency (EQE_PV_) spectra, photothermal deflection spectroscopy (PDS), or admittance spectroscopy.^9^ For the GDM, sub-bandgap EQE_PV_ analysis and temperature-dependent charge transport measurements—particularly using the space-charge-limited current method—are employed to estimate *σ*_s_.^15–18^ Among these, EQE_PV_-based extraction of *E*_U_ is most common, relying on exponential fitting of the sub-gap region of the EQE_PV_ spectrum.^9,19–22^

Despite its popularity, the Urbach approach has several limitations in the context of organic solar cells. The sub-gap EQE_PV_ signal generally consists of three distinct features: excitonic absorption from the donor and/or acceptor material, CT absorption from the donor–acceptor complex, and absorption due to trap states.^17^ Fitting this complex spectral region with a single exponential slope likely oversimplifies the underlying physics. While PDS measurements on neat films can isolate excitonic contributions and thus provide a cleaner assessment of *E*_U_, the interpretation of Urbach energies remains problematic. In many cases, *E*_U_ values are close to thermal energy (*k*_B_*T*), regardless of the measurement technique,^9,19–24^ which raises questions about its sensitivity and utility as a measure of disorder for organic semiconductors.

Device-based methodologies are invaluable for correlating energetic disorder with performance metrics in OSCs, as they capture the interplay between donor/acceptor material properties and device operation.^5,6,8^ However, to disentangle the intrinsic effects of molecular structure on the morphology, a more focused approach is needed. To this end, we will focus here on neat thin films of NFAs as they are free from the complexities introduced by the donor–acceptor complex. This will make it possible to directly investigate how structural molecular differences influence film morphology and the resulting excitonic disorder. Using the neat thin film comes with an additional benefit as now temperature-dependent absorption and photoluminescence (PL) spectroscopy can be used for assessment instead of the more complex techniques described above.

In this work, we compare three distinct methodologies to assess excitonic disorder in six representative NFAs, using thin films to explore how molecular design impacts their morphological and energetic properties. The three approaches make use of the temperature dependence of optical features in absorption and PL spectra of the NFA thin films. In absorption, the temperature dependence of the linewidth is used to assess both static and dynamic excitonic disorder,^25^ however, our analysis reveals that the static disorder is overestimated. We regard this approach as most useful only when information on the dynamic disorder properties is needed. A similar approach can also be applied to the linewidth in PL, but due practical and theoretical concerns, this method is deemed unreliable. Finally, the third method makes use of the temperature dependence of the first peak energy in PL,^26^ with the caveat of only allowing determination of the static disorder. This approach is both accurate and flexible in use, leading us to conclude it is the best performing methodology for *σ*_s_ assessment. For strongly aggregating molecules where CT interaction play a dominant role in the neat material, such as for the popular NFA Y6,^27^ we find and scrutinize the decreased accuracy of all three methods. Finally, the analysis of the structural differences in the investigated NFAs leads to the conclusion that compounds with linear *n*-octyl side chains systematically obtain the lowest static excitonic disorders, thus, providing evidence that these chains induce improved molecular packing as compared to bulkier 2-ethylhexyl and phenylhexyl side chains.

## 2 Experimental section

### 2.1 Materials and sample preparation

Six NFAs were studied in this work: EH-IDTBR, O-IDTBR, ITIC, CO*i*8DFIC, IDIC-4Cl and Y6. See Fig. S1 (ESI†) for the chemical structures. For each compound, a thin film sample was fabricated by spin casting it from chloroform solution of about 10 mg ml^−1^ at 2000 RPM onto a borosilicate glass substrate. For EH- and O-IDTBR, two additional neat thin film samples were made, now using a 10 mg ml^−1^ solution in chlorobenzene (CB). Both NFAs were also blended with the polymeric donor P3HT at a 1 : 1 weight ratio to achieve a 10 mg ml^−1^ solution in CB. From this solution two blend thin films were fabricated by spin casting them at 2000 RPM. All films were annealed at a temperature of 130 °C for 15 min.

### 2.2 Temperature-dependent and room temperature optical spectroscopy

The steady state PL and absorption spectrum of each sample was measured in a liquid nitrogen gas-exchange cryostat (Oxford Instruments Optistat DN and ITC502 temperature controller). The PL was obtained by exciting the thin film with a 405 nm laser (Thorlabs NPL41C) and measuring the resultant emission using an Andor Kymera 328i Spectrograph equipped with a Si-CCD (Andor iDus 416 Series). Using a slightly altered setup, and exchanging the laser for a broad band white light source (Avantes Avalight-HAL-CALMini), the transmittance spectrum of each film was measured which, in turn, was transformed to an absorption spectrum using the Beer–Lambert law. Room temperature transmittance and reflectance spectra were obtained using an Agilent Technologies Cary 5000 UV-Vis-NIR spectrometer equipped with an integrating sphere (Agilent Technologies Internal DRA). To obtain the absorption spectra in solution phase, a dilute solution of each compound in chloroform was made in a high precision quartz cuvette (Hellma Analytics). Then, the same UV-Vis-NIR spectrometer (without integrating sphere) was used to measure the transmittance from which the absorption could be determined through the Beer–Lambert law.

## 3 Disorder assessment using optical spectra

### 3.1 Spectral fitting

In order to apply the disorder assessment methodologies, we require a reliable way of extracting parameters from the optical spectra. A Franck–Condon (FC) progression, with one or more high frequency vibrational modes, can be used for this purpose.^25,28,29^ We found that this method can be somewhat unreliable for thin film spectra, sometimes needing unrealistically high vibrational energies in order to get a satisfactory fit. To omit these intricacies and keep the focus on the disorder assessment, we opt for a Sum-of-Gaussians (SoG) fitting procedure with the following functional form:
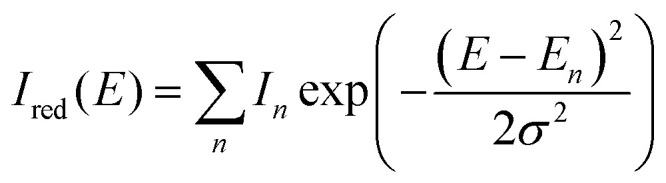


Here, *I*_red_(*E*) is the reduced spectrum as a function of the photon energy *E*, *σ* is the linewidth, and *E*_*n*_ and *I*_*n*_ are the energy and height of peak *n*. This SoG procedure is then used to fit either the reduced absorption spectrum *α*_red_ = *α*/*E* or the reduced PL spectrum *I*_PL,red_ = *I*_PL_/*E*^3^. Here, the reduced spectra (sometimes also referred to as lineshapes) are used to eliminate the respective linear and cubic dependence of the absorption and PL spectra on the photon energy *E*.^30^ Moreover, the PL has a cubic dependence on the photon energy only when expressed in counts per second (which are the units used for all PL spectra in this work), if it is expressed in Watts, the dependence becomes to the power of four. To be even more precise, a respective linear and cubic dependence on the refractive index *n*(*E*) should also be included in the absorption and PL spectra, but we assume it is practically constant across the investigated energy range. Finally, the results from the SoG procedure can be used to construct an effective FC model where the effective vibrational energy and Huang–Rhys (HR) factor are determined as *ħω*_eff_ = |*E*_0_ − *E*_1_| and *S*_eff_ = *I*_1_/*I*_0_, respectively.

### 3.2 Disorder assessment from absorption spectra

Analysing the temperature dependence of the absorption linewidth *σ* enables the extraction of static and dynamic excitonic disorder parameters in NFA thin films. This total linewidth *σ* has a contribution which is static *σ*_s_ and dynamic *σ*_d_ in nature resulting in the following decomposition:

1 *σ*^2^(*T*) = *σ*_s_^2^ + *σ*_d_^2^(*T*)

Here, the static contribution arises from the variation in molecular conformations and environment, resulting in a distribution on the singlet exciton energies. This distribution is assumed to be Gaussian in shape, in line with previous work, and is described by an excitonic DOS with standard deviation *σ*_s_ and mean energy *E*_gap_.^15,16,31^ Here, the term “static” points to the fact that *σ*_s_ has no temperature dependence (within the investigated range) describing variations in molecular conformation and environment which are essentially frozen solid at the time of deposition. The dynamic contribution in eqn (1) embodies the thermal motion of the molecules in the film and thus relates to low frequency vibrational modes (*ħω* < 0.05 eV). If one describes such low frequency vibrations using a FC progression, with the assumption of a large HR factor, one obtains an absorption spectrum which is approximately Gaussian in shape. This was first described by Thomas Keil, who derived the semi-classical formula for the variance *σ*_d,Keil_^2^ of such a Gaussian-like FC spectrum:^32^
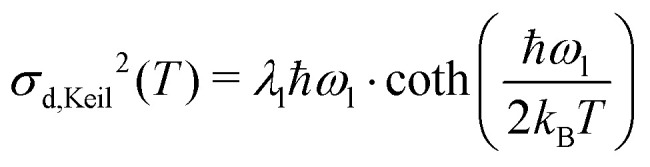


Here, *k*_B_*T* is the Boltzmann constant, *λ*_l_ is the low frequency reorganization energy, and *ħω*_l_ is the low frequency vibrational energy. In the high-temperature limit (*k*_B_*T* ≫ *ħω*_l_), the expression for *σ*_d_ simplifies to:*σ*_d,Marcus_^2^(*T*) = 2*λ*_l_*k*_B_*T*which is the same result one would find when describing the low frequency vibration classically, within the framework of Marcus theory.^33,34^ A more in-depth theoretical discussion on eqn (1) is included in ESI,† SI2.

In order to determine the static and dynamic excitonic disorder of a thin film sample, we first fit each spectrum in a set of temperature dependent absorption measurements using the SoG procedure. From this procedure, we will use the square of the total linewidth as a function of temperature, *σ*^2^(*T*), and fit it with eqn (1) where either Keil's or Marcus’ expression will be used for *σ*_d_^2^(*T*). The main difference between the two expressions lies in their low temperature behaviour, as for high temperatures *σ*_d,Keil_^2^ ≈ 2*λ*_l_*k*_B_*T*. The dynamic disorder, as described by the Marcus’ model, vanishes at *T* = 0 K, where *σ*_d,Keil_^2^ has a zero-point contribution equal to *λ*_l_*ħω*_l_. Consequently, at 0 K, the total squared linewidth *σ*^2^ will be equal to *σ*_s_^2^ in Marcus’ framework, while in Keil's framework it will be equal to *σ*^2^ = *σ*_s_^2^ + *λ*_l_*ħω*_l_. As a result, the static excitonic disorder extracted using Marcus’ framework will always be an overestimation due to the absence of this zero-point contribution. Thus, for the remainder of this study we will use *σ*_d,Keil_^2^ in our fitting procedures unless stated otherwise.

### 3.3 Disorder assessment from photoluminescence

**Fig. 1:**
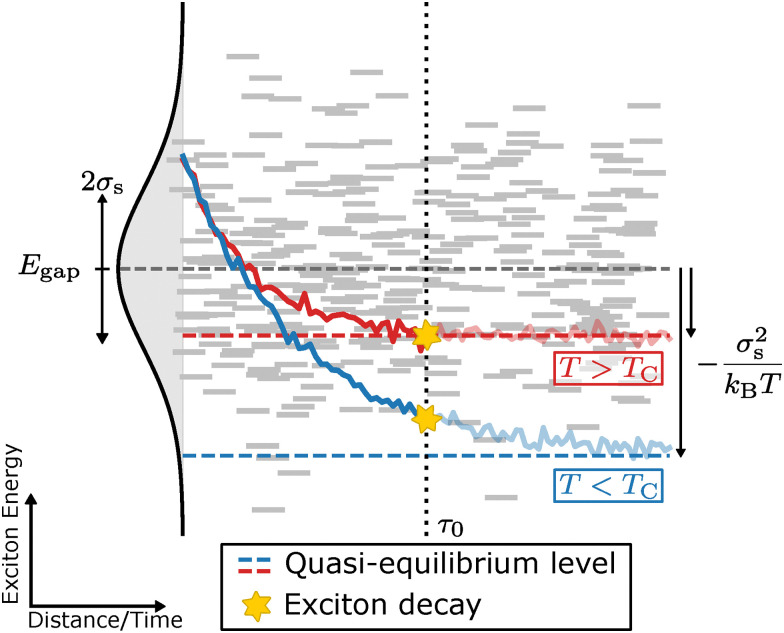
Schematic representation of the relaxation process of an exciton with lifetime *τ*_0_ in a Gaussian DOS with variance *σ*_s_ and mean energy *E*_gap_. For temperatures above the critical temperature *T*_C_, the exciton reaches quasi equilibrium before decaying (red curve). Below *T*_C_, the exciton relaxation time exceeds *τ*_0_, resulting in decay before quasi equilibrium is reached (blue curve).

The static excitonic disorder can also be assessed from PL as it is related to the bathochromic shift it experiences as a function of temperature.^26^ This shift is attributed to the exciton's diffusive behaviour, which allows for energetic relaxation: upon excitation, the exciton is able to perform a random walk through the molecular solid before decaying. Initially, this migration follows an energetically downhill path, after which the exciton reaches a quasi-equilibrium between downward and thermally activated upward jumps. At that point, the most populated energy level lies at *σ*_s_/*k*_B_*T* below the center of the (previously described) Gaussian DOS, at which the exciton decays resulting in peak fluorescence at an energy equal to *E*_gap_ − *σ*_s_/*k*_B_*T*.^11^ This process of migratory exciton relaxation is referred to as spectral diffusion. When the temperature is decreased, quasi-equilibrium will be reached at lower and lower energy levels. As a result, the time it takes for the exciton to reach this quasi-equilibrium increases with decreasing temperature. At some critical temperature, *T*_C_, this relaxation time will exceed the lifetime of the exciton (*τ*_0_) at which point the exciton will decay before reaching quasi-equilibrium (see Fig. 1 for a schematic representation of this process). Consequently, below *T*_C_, the bathochromic shift of the fluorescence peak starts to stagnate, deviating from the *E*_gap_ − *σ*_s_/*k*_B_*T* trend.^26^

A SoG fit of each spectrum in a set of temperature dependent PL measurements will be the first step for determining the static disorder of a thin film sample. Then, a linear fit is performed on the energy of the first peak *E*_0_ as a function of inverse temperature *T*^−1^ for temperatures above *T*_C_. Finally, we extract *σ*_s_ from the slope of this linear fit, which is equal to *σ*_s_^2^/*k*_B_*T*. The critical temperature is determined as being the temperature at which *E*_0_(*T*^−1^) starts to deviate from linearity.

An approach involving the temperature dependence of the square of the PL linewidth *σ*^2^(*T*), analogue to the one outlined in the previous section for absorption spectra, could in principle also be applied to PL spectra. Though, there is one major difference between the nature of the static component of the linewidth in absorption and PL. In absorption, the total width of the excitonic DOS makes up the static component in eqn (1). In PL, the exciton first relaxes before decaying, and thus probes the occupational excitonic density of states (ODOS). In the quasi-equilibrium regime the variance of the ODOS is the same as the DOS, *i.e. σ*^ODOS^_s_ = *σ*^DOS^_s_ for *T* > *T*_C_.^11,26^ For temperatures below *T*_C_ this can no longer be guaranteed.^25^ Consequently, we expect the static contribution in eqn (1) for PL to no longer be completely “static” in nature for *T* < *T*_C_. Thus, only data above the critical temperature is to be used when applying *σ*^2^(*T*) fitting to PL data. Furthermore, since Marcus’ and Keil's expression for *σ*_d_ only differ in the low temperature regime, and since *σ*_d,Keil_^2^ ≈ 2*λ*_l_*k*_B_*T* at higher temperatures, only *σ*_d,Marcus_^2^ is to be used for fitting above the critical temperature to prevent excessive parameter freedom. Consequently, the extracted static excitonic disorder is expected to be overestimated due to the lacking zero-point contribution.

### 3.4 Limitations of the Gaussian disorder model

The working principle of all the methods employed in this study begins with the assumption of a Gaussian excitonic DOS, where the distribution width reflects variations in molecular conformation and environment. In essence, we assume that all morphological complexity of the system can be fully captured by treating excitonic energies as normally distributed. Intermolecular interactions are thus treated implicitly. However, they do play a crucial role in organic semiconductor solids and account for the distinct differences observed between solution-phase and thin-film optical spectra.^35^

The objective of this work is not to explore the impact of nanoscopic aggregation effects on spectral shape—an area that has been extensively studied in the literature.^27,35,36^ Instead, we adopt a top–down approach to assess how morphological insights can be inferred from the temperature dependence of optical spectra in NFA thin films. We thus assume that the temperature dependence of these optical features is negligibly influenced by intermolecular interaction, allowing for determination of global disorder parameters. However, the implicit treatment of intermolecular interactions in our models carries an important caveat: vibrational parameters extracted from thin film data *via* the SoG procedure (*ħω*_eff_ and *S*_eff_) should be regarded as pseudo-parameters and should only be used for comparison of spectral shapes. Since thin film spectra are shaped by aggregation effects, these parameters no longer correspond solely to intrinsic molecular vibrations but instead reflect a convolution of both intra- and intermolecular influences.

## 4 Results and discussion

### 4.1 General results and methodology overview

**Fig. 2:**
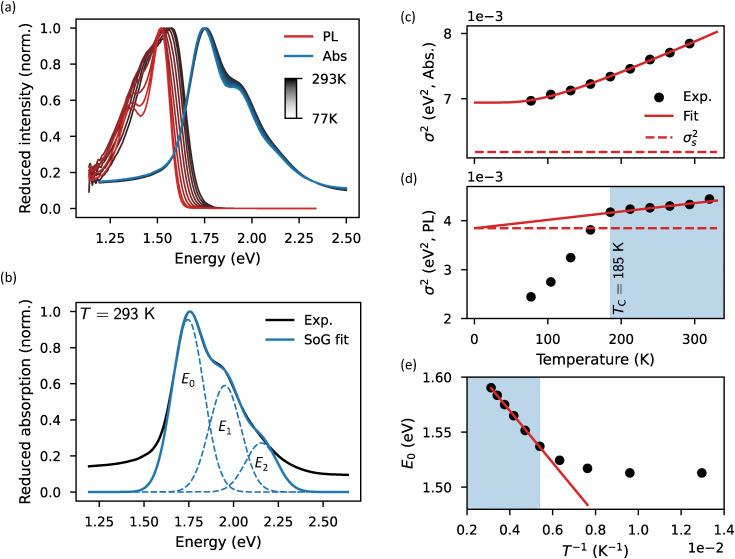
(a) Reduced and normalized temperature dependent PL (red) and absorption (blue) spectra of ITIC in thin film setting. The temperature difference between two consecutive spectra is 27 K, going from 293 K to 77 K. (b) SoG fit (blue) of the room temperature reduced absorption spectrum (black) of the ITIC thin film sample. Square of the linewidth of the absorption (c) and PL (d) as a function of temperature for the ITIC thin film sample fitted with eqn (1) using Keil's and Marcus’ expression for *σ*_d_^2^, respectively. The dashed line indicates the contribution from the static excitonic disorder. (e) First peak energy of the PL spectrum as a function of inverse temperature *E*_0_(*T*^−1^), obtained from the SoG procedure, linearly fitted for *σ*_s_ (red line). Only PL data with temperatures above *T*_C_ (light blue area in (d) and (e)) are used for disorder assessment.

To evaluate the effectiveness of the methodologies for quantifying excitonic disorder, we characterized the temperature-dependent absorption and PL spectra of six NFAs in their thin film form (details in the Experimental section). Fig. 2 shows the steps that are undertaken in the application of the methodologies where the characterization of ITIC was chosen as an example. The first two steps are practically the same for absorption and PL: measure the spectra as a function of temperature (Fig. 2a) and fit each reduced spectrum with the SoG procedure (Fig. 2b). Then, for absorption, the square of the linewidth as a function of temperature *σ*^2^(*T*) is fit with eqn (1) where Keil's expression for *σ*_d_^2^ is used (Fig. 2c). For PL, the same procedure is applied: the square of the linewidth as a function of temperature *σ*^2^(*T*) is fit with eqn (1), but now incorporating Marcus’ expression for *σ*_d_^2^, and only for data above *T*_C_ (Fig. 2d). Finally, for PL, the first local peak energy as a function of inverse temperature *E*_0_(*T*^−1^) is linearly fit using *E*_gap_ − *σ*_s_^2^/*k*_B_*T* (Fig. 2e). The room temperature absorption spectra of the materials in chloroform solution were also measured and analysed with the SoG approach for comparison (see ESI,† SI3). All the relevant extracted data is summarized in Table 1. The temperature dependent absorption and PL spectra for all materials, together with their respective disorder extraction fits, are presented in Fig. S4–S7 (ESI†).

**Table 1:** Solution to film shift and overview of the extracted disorder parameters of the six investigated NFAs. Solution to film shift is determined as Δ*E*^(sol–film)^_0_, with *E*^(sol)^_0_ and *E*^(film)^_0_ the first absorption peak energy of the material in dilute chloroform solution and thin film setting, respectively

Molecule	From absorption					From photoluminescence			
	Δ*E*^(sol–film)^_0_^a^ (meV)	*σ* ^2^(*T*) fit				*E*_0_(*T*^−1^) fit	*σ*_2_ (*T*) fit		
		*σ*_s_ b (meV)	*σ*_d_ a b (meV)	*λ*_l_ b (meV)	*ħω*_l_ b (meV)	*σ*_s_ (meV)	*σ*_s_ c (meV)	*σ*_d_ a c (meV)	*λ*_l_ c (meV)
EH-IDTBR	61.2	73.61	60.28	65.03	28.80	41.87	59.31	26.26	13.64
O-IDTBR	183.5	50.90	73.92	90.47	39.44	31.80	22.86	55.73	61.48
ITIC	74.4	78.63	40.74	30.42	24.91	45.08	62.00	22.77	10.26
CO*i*8DFIC	129.2	84.98	42.22	32.69	24.81	41.40	43.04	46.69	43.15
IDIC-4Cl	177.6	51.80	38.00	26.23	26.39	25.22^d^	0.60	38.74	29.70
Y6	214.4	92.14	0.06	0.04		44.12	0.14	66.88	88.54

a Determined at 293 K.

b Disorder parameters obtained with Keil's expression for *σ*_d_^2^.

c Disorder parameters obtained with Marcus’ expression for *σ*_d_^2^.

d *σ*_s_ determined using *E*_p_ instead of *E*_0_ (see Section 4.3).

Before studying specific cases, we first take note of some general trends in the data. Firstly, the static excitonic disorder *σ*_s_ obtained from absorption is 20–40 meV higher in magnitude than *σ*_s_ obtained from PL. We attribute this discrepancy to the experimental method used for obtaining the absorption spectrum. Here, the transmittance of the thin film is measured and transformed using the Beer–Lambert law. This is not strictly correct: also reflectance and interference effects should be considered. However, the measurement of reflectance in a cryostat is practically difficult. Omitting the reflectance in the determination of the absorption has several effects on the absorption lineshape (see ESI,† SI5). The most important effect for our study is that the linewidth (*σ*) is overestimated by, on average, 14 meV when the reflectance is omitted in the Beer–Lambert law. The discrepancy in static excitonic disorder observed for *σ*^2^(*T*) fitting in absorption stems directly from this effect. Specifically, an overestimation of the linewidth (*σ*) will result in an overestimation of the extracted *σ*_s_. Thickness dependent interference effects, on the other hand, have little to no effect as the additional error induced, here, is largely overshadowed by the error induced by the omittance of the reflectance in the Beer–Lambert law (ESI,† SI6).

For PL, disorder assessment is not only affected by interference, but also by self-absorption of the emitted light.^37,38^ As detailed in ESI,† SI7, we estimate these effects to negligibly impact the extracted static excitonic disorder while resulting in an approximate 50 meV underestimation of the mean gap energy *E*_gap_.

Now, it is worth noting that disorder extraction from the temperature dependence of the PL peak energy is more flexible in use than disorder extraction from the temperature dependence of the absorption and PL linewidth. Specifically, if the SoG method for any reason fails to provide a satisfactory fit, disorder assessment through the analysis of the linewidth of the spectrum becomes impaired. In PL, *σ*_s_ assessment can still be achieved through the temperature dependence of the experimental peak energy *E*_p_(*T*^−1^), albeit with some accuracy loss. Here, the experimental peak energy *E*_p_ refers to the energy at which the reduced PL spectrum is maximal while *E*_0_ refers to the energy of the first peak extracted from the SoG procedure. Notice that *E*_0_ and *E*_p_ do not necessarily coincide (Fig. 2b). Here, overlap between *E*_0_ and *E*_1_ in the SoG fitting procedure tends to push *E*_0_ away from the experimental maximum *E*_p_.

When taking a further look at the data in Table 1, we notice that the relative static excitonic disorder differences between the different materials, acquired from of *σ*^2^(*T*) fitting in absorption and *E*_0_(*T*^−1^) fitting in PL, broadly seem to agree with each other. In contrast, the data obtained from *σ*^2^(*T*) fitting in PL proved to be inconsistent, unrealistically predicting vanishing static excitonic disorders for IDIC-4Cl and Y6. This, in combination with the theoretical complications discussed in Section 3.3, leads us to conclude that assessing disorder parameters by fitting *σ*^2^(*T*) in PL is significantly less reliable than using any of the other two methodologies for this purpose. Consequently, we will omit the results from *σ*^2^(*T*) fitting of PL spectra from the discussions below.

Finally, the values of *σ*_s_ obtained for ITIC, EH-IDTBR, and Y6 through *E*_0_(*T*^−1^) fitting of the PL peak energies are generally consistent with those reported in the literature using a similar approach.^39^ However, due to a slightly different application of the method, the results are not directly comparable.

Ultimately, while the trends observed from *σ*^2^(*T*) fitting in absorption and *E*_0_(*T*^−1^) fitting in PL broadly align, the assessment of static excitonic disorder is most reliable when using *E*_0_(*T*^−1^) fitting in PL, as it is free from the inherent practical and theoretical issues associated with the other two methodologies.

### 4.2 The effect of side chain variation

**Fig. 3:**
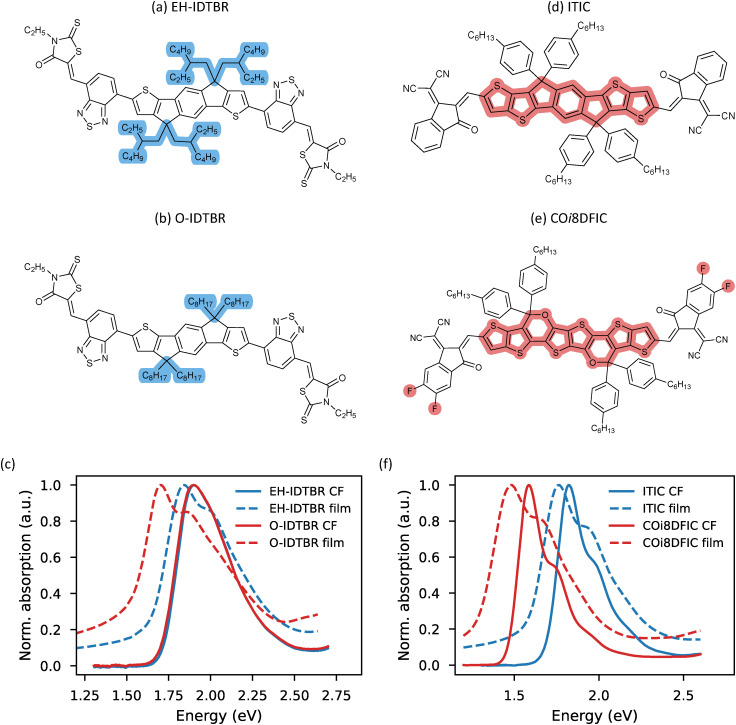
The chemical structures of EH-IDTBR (a) and O-IDTBR (b) with the highlighted areas indicating the structural differences. Their normalized absorption spectra in dilute chloroform solution and thin film setting are shown in (c). The chemical structures of ITIC (d) and CO*i*8DFIC (e) with the highlighted areas indicating the structural differences. Their normalized absorption spectra in dilute chloroform solution and thin film setting are shown in (f). All spectra were measured at 293 K. For chemical structures without highlighting see Fig. S1 (ESI†).

Fig. 3a and b show the chemical structures of EH-IDTBR and O-IDTBR, respectively. The primary difference between these molecules lies in their side chains: EH-IDTBR has branched 2-ethylhexyl side chains, while O-IDTBR has linear *n*-octyl side chains. Their solution-phase absorption spectra are identical (Fig. 3c), as expected given their identical core moiety. Upon transitioning to the film state, a redshift is observed in the absorption spectra, with O-IDTBR and EH-IDTBR shifting by 184 and 61 meV, respectively. This difference arises from the notable impact of side chain variation on the aggregation properties of the IDT-BT core. Indeed, Holliday *et al.* demonstrated that O-IDTBR exhibits a higher degree of crystallinity compared to EH-IDTBR.^40^ Here, the linear *n*-octyl side chains were found to promote better molecular packing compared to the branched 2-ethylhexyl chains. The results obtained from absorption and PL show a similar trend where O-IDTBR has a static excitonic disorder which is 25–35% smaller than the static disorder of EH-IDTBR (Table 1). For the (room temperature) dynamic excitonic disorder, the roles are reversed where *σ*_d_ is larger in magnitude for O-IDTBR than for EH-IDTBR. This increase in dynamic disorder for O-IDBTR stems from an increase in both *λ*_l_ and *ħω*_l_. Thus, the difference in side chains not only affects the packing properties of the materials but also seems to alter the low frequency vibrational properties of the compounds in the thin film setting.

Fig. 3d and e depict the chemical structures of the acceptor–donor–acceptor (A–D–A) type NFAs ITIC and CO*i*8DFIC, respectively. Unlike the previous case, both compounds share identical hexylphenyl side chains but differ in their core moieties. Specifically, ITIC features a seven-membered indacenodithiophene (IDT) donor core, while CO*i*8DFIC incorporates an oxygenated eight-membered donor core. Additionally, both molecules possess indenecyanovinylene (IC) acceptor units, which in the case of CO*i*8DFIC are fluorinated. The absorption spectra of the two compounds in dilute chloroform solution, shown in Fig. 3f, reveal that CO*i*8DFIC is redshifted relative to ITIC. This redshift arises from the more electron-donating nature of the CO*i*8DFIC donor core, combined with the fluorination of its acceptor blocks.^41^ However, the difference in core moiety primarily influences the gap energy, as the lineshapes of the two compounds’ solution spectra are highly similar. This observation is further supported by the effective FC parameters obtained from a SoG fit of these spectra (Table S1, ESI†), which shows that both spectra are dominated by a vibrational mode with an energy of approximately 165 meV and a HR factor of 0.47.

Upon transitioning from solution to thin film, the absorption spectra of CO*i*8DFIC and ITIC exhibit redshifts of 129 meV and 74 meV, respectively. Despite this difference in redshift magnitudes, the absorption lineshapes of both materials in the thin film state remain strikingly similar (Fig. 3f). This similarity is further reflected in their effective FC (pseudo-)parameters, with both materials exhibiting *ħω*_eff_ ≈ 210 meV and *ħω*_eff_ ≈ 0.63 (Table S1, ESI†). It is important to note that these effective FC parameters, when applied to thin film spectra, serve only as pseudo-parameters and are used here solely for comparative analysis of spectral shapes.

The consistency between the two compounds further extends to their energetic disorder. The static and dynamic excitonic disorder parameters extracted from *σ*^2^(*T*) fitting in absorption, as well as the static excitonic disorder derived from *E*_0_(*T*^−1^) fitting in PL, were found to be in close agreement. This suggests that both thin films exhibit comparable degrees of disorder, despite differences in their core structures. Moreover, the variation in core units provides an explanation for the disparity in solution-to-film redshift observed between CO*i*8DFIC and ITIC. While aggregation effects contribute to these shifts—likely to a similar extent in both materials—another key factor is the molecular response to changes in the dielectric environment when transitioning from solution to the solid state.^27^

Ultimately, these results demonstrate the substantial impact of side chain selection on the energetic disorder, and consequently, on the film morphology.

### 4.3 Evaluation of the robustness of the methodologies

In order to assess the overall reliability of optical fitting methods, it is important to discuss the edge cases where difficulties were encountered. Recall that our methodologies are built up of two steps: we first fit the temperature dependent spectra of a thin film sample with the SoG method, where after the extracted optical features are used for assessing the excitonic disorder. The problem with this approach is that disorder assessment becomes impaired when the SoG procedure results in a less than optimal fit. SoG fitting difficulties mainly occurred for IDIC-4Cl and Y6 (see Fig. S1, ESI,† for chemical structures), with varying severity.

**Fig. 4:**
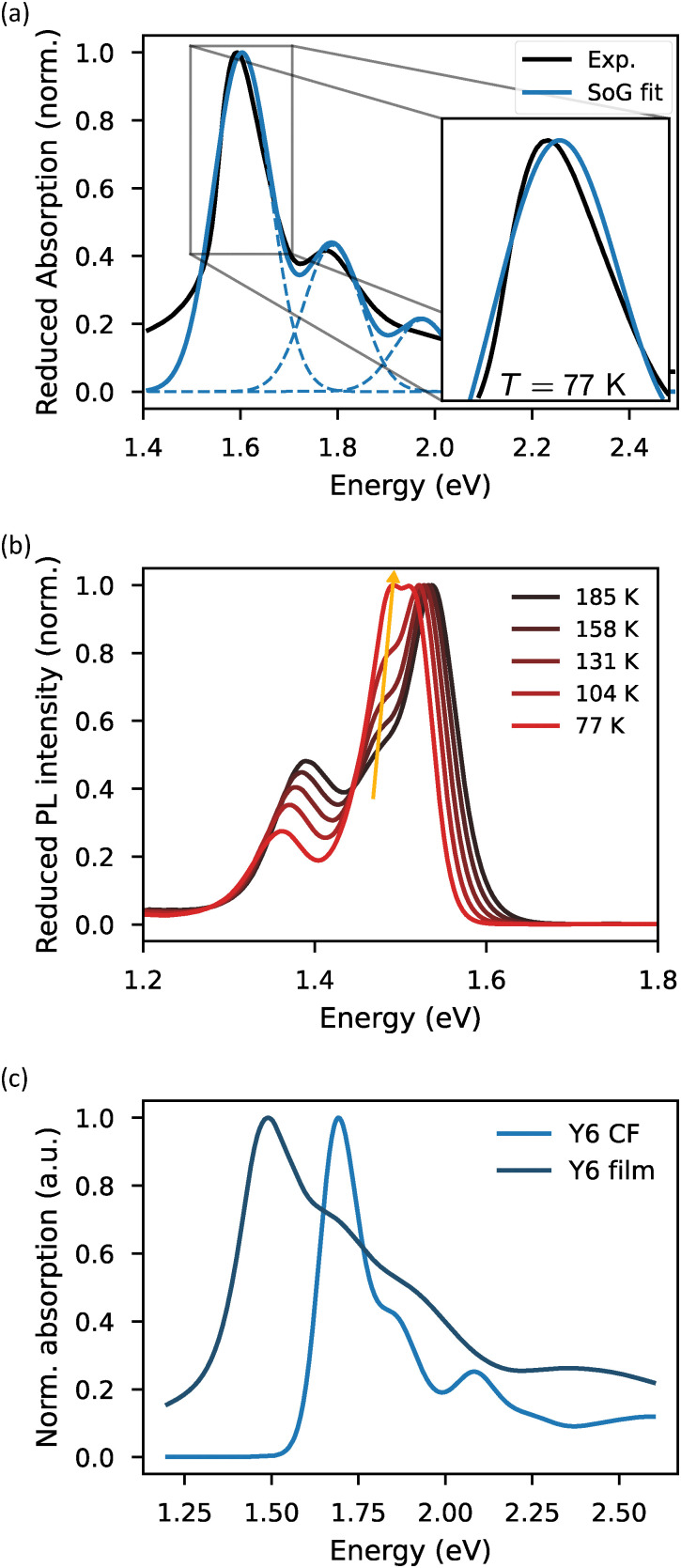
(a) SoG fit (blue) of the 77 K reduced absorption spectrum (black) of IDIC-4Cl in thin film setting. The inset shows the asymmetry of the first absorption peak. (b) Temperature dependent reduced PL spectrum of IDIC-4Cl in thin film setting. The yellow arrow indicates the emergence of a second intermediate vibronic peak. (c) Room temperature absorption spectra of Y6 in dilute chloroform solution and thin film phase.

For IDIC-4Cl, the main problem is that the onset peak of the absorption spectra is no longer exactly Gaussian and starts to become slightly asymmetric (Fig. 4a). This deviation from the Gaussian shape slightly reduces the accuracy of both the extracted first peak energies and linewidths. Additionally, the fit quality in the higher-energy region of the spectrum is also suboptimal. However, since the primary objective of the SoG method is to capture the linewidth variation of the lowest-energy peak, discrepancies at higher energies are less critical. This is because both static and dynamic disorder broaden the entire spectrum uniformly, meaning that accurately fitting the first peak provides sufficient information for the subsequent disorder analysis.

The source of the asymmetry of the first absorption peak can be uncovered when taking a closer look at the temperature dependent PL spectra in the thin film setting (Fig. 4b). Here, a second vibronic peak emerges next to the onset peak as the temperature decreases. Now, when moving from PL to absorption, the spectrum of IDIC-4Cl increases slightly in width. We thus expect the asymmetry of the first absorption peak to be coming from this intermediate vibrational mode which cannot be discerned due to the broader nature of the absorption spectrum. That being said, the SoG fitting issues are only minor, still allowing for assessment of the excitonic disorder from the temperature dependent absorption measurements. The emergence of the intermediate vibrational peak in the temperature-dependent PL spectrum also negatively affects the SoG fit quality here. However, as discussed in Section 4.1, the temperature dependence of the experimental peak energies (*E*_p_) can be used for disorder assessment in such cases, instead of relying on the fitted first peak energies (*E*_0_) from the SoG procedure. The case of IDIC-4Cl thus highlights the greater flexibility of the PL redshift method for evaluating static excitonic disorder. Furthermore, the results for IDIC-4Cl actually show a similarly low value for the static excitonic disorder as O-IDTBR, which possesses the same *n*-octyl side chains. This result thus seems to reaffirm that the *n*-octyl side chains promote lower static excitonic disorder. Finally, temperature-dependent spectral changes in PL are not unique to only IDIC-4Cl. As shown in Fig. S4 (ESI†), nearly all investigated NFAs exhibit variations in PL lineshape with temperature. However, only in the case of IDIC-4Cl did these changes significantly interfere with the SoG fitting procedure. Such spectral evolution arises from spectral diffusion processes occurring prior to emission, where excitons relax into progressively lower energy states within the DOS. These states can be conformationally, environmentally and energetically different from the states laying closer to the DOS center. This can, in turn, lead to spectral lineshapes deviating from the room temperature shape as emission now comes from only a small portion of all possible states. For IDIC-4Cl, in particular, the hypothesis is that these lower energy excitons exhibit emission with a higher intensity of the observed intermediate vibration.

For Y6, the SoG fitting problems are mainly caused by the significant broadening of the thin film absorption spectrum beyond the first peak, at photon energies above 1.6 eV (Fig. 4c). This poses a challenge for the SoG procedure as, here, every peak is assumed to have the same linewidth. In addition to the SoG fitting problems, assessment of excitonic disorder in Y6 proves to be particularly problematic. Here, *σ*^2^(*T*) fitting in absorption predicts a vanishing low frequency reorganization energy with a static excitonic disorder of 92 meV, which is the largest value we have encountered in this work. Furthermore, *E*_0_(*T*^−1^) fitting in PL predicts a static excitonic disorder of 44 meV, which again is on the larger side (for this method). This observation is very counterintuitive as Y6 displays characteristics of a material with very strong aggregational properties. This, in turn, would result in a highly crystalline film and, consequently, a very low expected static disorder, contradicting the high static excitonic disorders we obtained.

The explanation for this inconsistency lies with the assumptions that are made in the disorder assessment methodologies. More specifically, we assume that the temperature dependence of the optical features used for disorder assessment are only negligibly influenced by intermolecular interactions. The study of Y6 is a clear example where this assumption breaks down. Y6 is known to have very strong intermolecular interactions in the thin film setting, allowing not only for localized excitons, but also for CT excitations.^42^ Together, these localized and CT excitations greatly impact the absorption features of Y6 in the thin film setting, resulting in a broadening behaviour greatly influenced by intermolecular interactions.^27^ Even though no SoG fitting difficulties were encountered for the PL spectra, it is difficult to deem these results trustworthy.

The Y6 case study thus shows that once CT interactions become significant, the applicability of these disorder assessment methodologies is fundamentally compromised. Therefore, when CT interactions play a dominant role in the system under investigation, caution is warranted, and alternative approaches should be considered for characterization of the energetic disorder.

### 4.4 The link with organic semiconductor devices

A natural question arising from the results in Table 1 is whether the static excitonic disorder parameters correlate with OSC performance. Specifically, does a lower degree of static excitonic disorder translate into improved device efficiency?

Unfortunately, this question does not have a straightforward answer. Device performance is determined by several interdependent factors. First, the choice of donor material plays a critical role and often varies for each NFA, making direct comparison challenging. Differences in the donor–acceptor energy level alignment—particularly the HOMO and LUMO offsets—affect the open-circuit voltage (*V*_OC_). Additionally, the absorption profiles of different NFAs span distinct spectral regions, resulting in distinct differences in EQE_PV_ and hence the short-circuit current density (*J*_SC_).

Given these factors, establishing a direct correlation between static excitonic disorder and the power conversion efficiency (PCE) is complicated. The fill factor (FF), however, is more closely related to charge transport properties such as hole and electron mobilities, and may therefore be more sensitive to underlying disorder.^7^ Still, it is important to note that the disorder parameters presented here were extracted from neat NFA thin films. When blending the NFAs with a donor material, the morphology—and by extension, the degree of disorder—can change significantly.

**Fig. 5:**
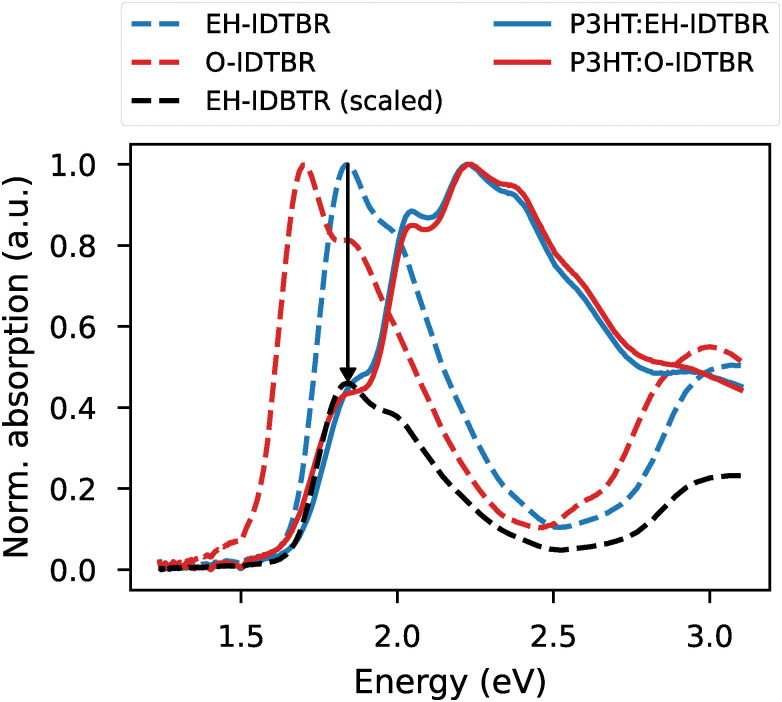
Normalized absorption spectra of neat EH-IDTBR and O-IDTBR thin films (dashed lines), and their 1 : 1 blends with the polymer donor P3HT (solid lines). A scaled spectrum of EH-IDTBR (×0.5) is included in black for comparison.

This point is illustrated in Fig. 5, which compares the absorption spectra of neat EH-IDTBR and O-IDTBR films with those of their respective blends with P3HT at a 1 : 1 weight ratio. In the neat film state, the absorption of O-IDTBR is clearly redshifted relative to EH-IDTBR, reflecting differences in aggregation driven by side chain variation. Upon blending with P3HT, these spectral differences largely disappear, suggesting that the donor polymer influences the acceptor's aggregation behaviour—and thus its energetic disorder. In fact, the acceptor absorption in both blends closely resembles that of neat EH-IDTBR, implying that the improved aggregation observed in neat O-IDTBR is suppressed in the blend. This interpretation is supported by device data from Holliday *et al.*, who reported nearly identical FFs and electron mobilities for EH- and O-IDTBR when blended with P3HT in a 1 : 1 weight ratio.^40^ As such, the static disorder values reported in Table 1 likely represent lower limits, as blending with a donor is expected to increase disorder due to morphological disruption.

Finally, a more direct comparison can be made with mobilities measured in organic thin-film transistors (OTFTs), where NFAs are studied in their neat form. Bristow *et al.* reported n-type saturation mobilities for O-IDTBR, EH-IDTBR, and ITIC of 0.12, 0.06, and 0.01 cm^2^ V^−1^ s^−1^, respectively.^43^ These values show an inverse correlation with the static disorder parameters reported here, with O-IDTBR showing the lowest disorder and highest mobility, and ITIC exhibiting the highest disorder and lowest mobility. This trend supports the idea that reduced static excitonic disorder may be associated with improved charge transport properties in neat films.^44,45^

## 5 Conclusions

This work presents three approaches for quantifying static and dynamic excitonic disorder in organic semiconductors by analysing the temperature dependence of spectral features in thin film absorption and PL spectra. For absorption, the excitonic disorder parameters—static disorder, low-frequency reorganization energy, and low-frequency vibrational energy—are extracted from the temperature dependence of the linewidth. However, the static disorder parameter, *σ*_s_, is likely overestimated using this method. While a similar approach can be applied to the PL linewidth, it is strongly discouraged due to inconsistencies in the extracted disorder parameters and theoretical complications. Alternatively, fitting the temperature dependence of the first PL peak energy was found to be the most suitable and reliable method for extracting static disorder, with the added benefit of being flexible in application.

Using these methodologies, three case studies are undertaken. First, EH- and O-IDTBR are analysed, revealing that the linear *n*-octyl side chains of O-IDTBR promote lower static excitonic disorder than the bulkier 2-ethylhexyl chains of EH-IDTBR in the neat thin film setting. The second case study compares CO*i*8DFIC and ITIC, which share phenylhexyl side chains but differ in their core moiety, leading to similar static disorder, further emphasizing the impact of side chains on packing behaviour. Finally, difficulties were encountered in the characterization of IDIC-4Cl and Y6, with differing severity. IDIC-4Cl, with *n*-octyl side chains like O-IDTBR, exhibited low disorder, though asymmetric spectral features reduced fitting accuracy. Y6, known for strong intermolecular interactions, unexpectedly showed one of the highest static excitonic disorder values, highlighting the need for caution when interpreting disorder metrics in materials where charge-transfer intermolecular interactions play a dominant role. Finally, the static excitonic disorder values reported here were obtained from neat NFA thin films and likely represent lower bounds. Spectral shifts observed upon blending with donor polymers suggest changes in aggregation behaviour that may increase disorder. This highlights the importance of blend morphology when linking disorder to device performance metrics like the fill factor. Additionally, comparison with OTFT mobility data reveals an inverse correlation between disorder and charge transport, suggesting that reduced static disorder may support improved carrier mobility.

Together, these insights provide valuable guidance for both the analysis of excitonic disorder and the molecular design of organic semiconductors, paving the way for further improvements in material morphology and device performance.

## Author contributions

Siebe Frederix: data curation, investigation, methodology, software, writing – original draft, writing – review & editing. Samuele Giannini: writing – review & editing. Melissa Van Landeghem: writing – review & editing. David Beljonne: supervision, writing – review & editing. Koen Vandewal: funding acquisition, supervision, writing – review & editing.

## Conflicts of interest

There are no conflicts to declare.

## Supplementary Material

MH-012-D5MH00547G-s001

## Data Availability

The data supporting this article have been included in the manuscript and as part of the ESI.†
