# Supplementary material for: Extracting disorder parameters from optical spectra of non-fullerene acceptors
Source: Mater Horiz. 2025 Jul 7;12(19):8048–58. doi: 10.1039/d5mh00547g (PMC12231089; doi:10.1039/d5mh00547g)
Supplement: MH-012-D5MH00547G-s001 [file MH-012-D5MH00547G-s001.pdf]

Supplementary Information (SI) for Materials Horizons.
This journal is © The Royal Society of Chemistry 2025

Supplementary Information

Extracting disorder parameters from optical spectra of non-fullerene acceptors

Siebe Frederix, Samuele Giannini, Melissa Van Landeghem, David Beljonne, Koen Vandewal**

SI 1. Chemical structures

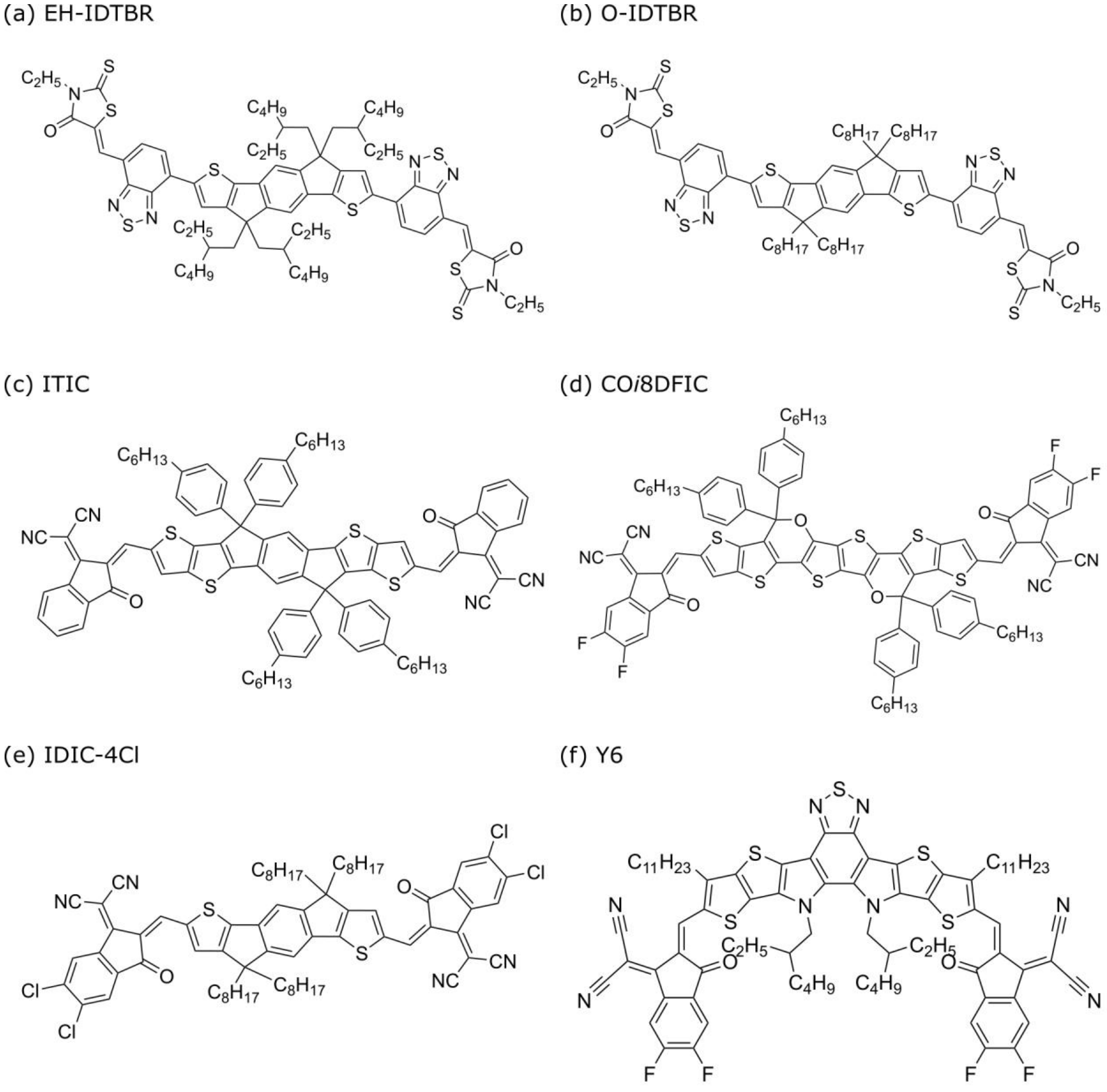

Figure S1. Chemical structures of a) EH-IDTBR, b) O-IDTBR, c) ITIC, d) CO*i*8DFIC, e) IDIC-4Cl, and f) Y6.

SI 2. Theoretical background on absorption spectrum modelling of thin films

In order to give a comprehensive derivation/explanation on the role of disorder in thin film absorption spectra of organic semiconductors, it is easier to start with the absorption spectrum of a single molecule before extending our analysis to thin films. An absorption event for a single molecule is a transition from the molecule's singlet ground state to a singlet excited state through the absorption of a photon.[1,2] The singlet ground state and the singlet excited state are described by two potential energy surfaces, which represent the energy landscape of the states as a function of the molecule's reaction coordinates (*e.g.* bond lengths, bond angles, …). For small displacements, these surfaces can be approximated by parabolas which describe a set of uncoupled quantum harmonic oscillators when treated in normal mode coordinates. When solved, the solutions of this quantum system describe quantized modes, representing vibrations of the molecule. The molecule dependent curvature and horizontal displacement of these harmonic energy surfaces determine the vibrational energy and the reorganization energy of the vibrational mode. Thus, the energy landscape of a molecule, in the

harmonic approximation, can be described as a set of displaced harmonic oscillators, each subdivided into quantized vibronic energy levels (Figure S2a). An absorption event, thus, corresponds to a transition from a vibrational energy level in the electronic ground state to a vibrational level in the electronic excited state. The strength of this transition is dictated by the transition dipole moment (or the related oscillator strength) of the electronic transition and the vibrational wavefunction overlap between the ground and excited states. However, not all vibrational levels of the ground state are involved in the transition. The molecule is in thermal equilibrium and thus the ground state vibrational levels are thermally populated according to a Boltzmann distribution. Consequently, the bulk of the absorption transitions originate from the lowest vibrational level in the electronic ground state.

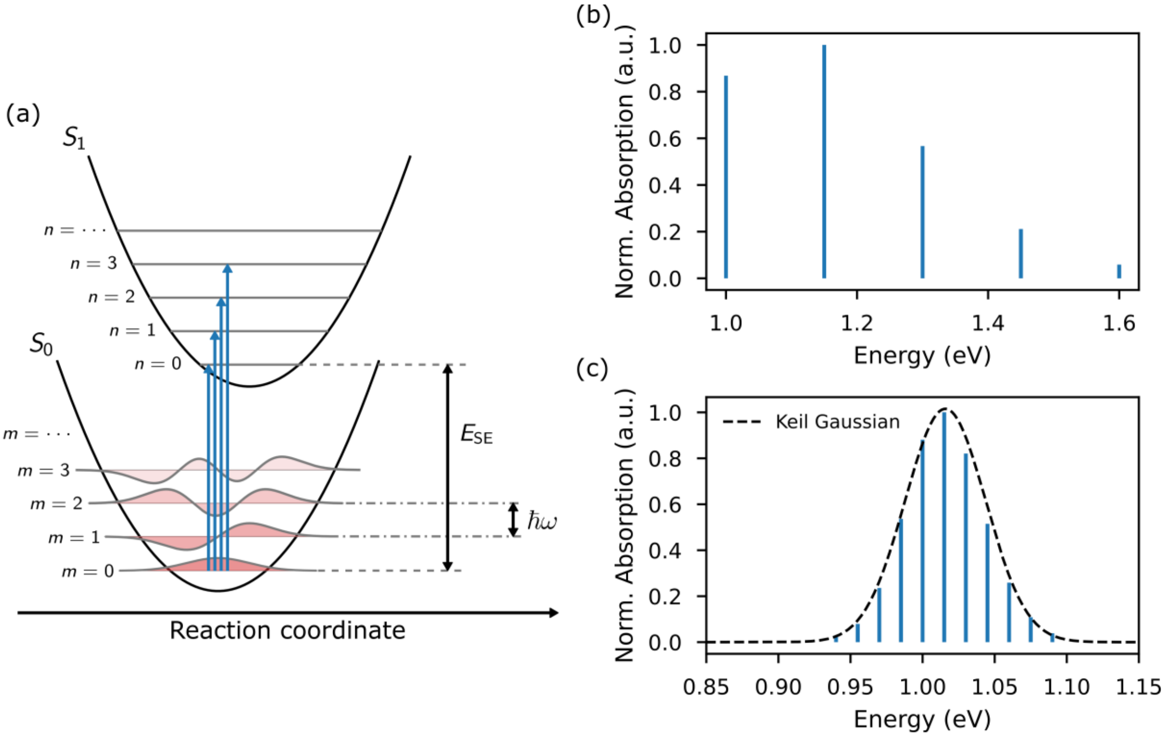

Figure S2. a) Potential energy diagram as a function of the reaction coordinate showing the possible vibrational transitions in an excitonic system with one effective vibrational mode, having vibrational energy $\hbar\omega$ and singlet exciton energy E_{SE}. b) Absorption spectrum calculated with Equation S1 with $\hbar\omega = 0.15$ eV, $S = 1$, and $E_{\mathrm{SE}} = 1$ eV. c) (blue) Absorption spectrum calculated with Equation S1 with $\hbar\omega = 0.015$ eV, $S = 1$, and $E_{\mathrm{SE}} = 1$ eV. (dashed line) Gaussian curve calculated with Keil's approximation using $E_{\mathrm{SE}} = 1$ eV, $\hbar\omega = 0.015$ eV, and $\lambda_{\mathrm{l}} = 0.015$ eV. All spectra are calculated at $T = 293$ K.

To determine the absorption spectrum for a single molecule with one vibrational mode in vacuum, three parameters are required: the singlet exciton energy E_{SE}, which describes the energy difference between the electronic ground and excited states; the vibrational energy $\hbar\omega$; and the vibrational reorganization energy λ, related to the Huang-Rhys (HR) factor $S = \lambda/\hbar\omega$, describing the coupling strength between vibrational levels. Generally, a molecule with N_{a} atoms will have $3N_{\mathrm{a}} - 6$ vibrational modes (if not linear), where each additional vibrational mode introduces a vibrational energy $\hbar\omega$ and an associated HR factor S. For NFAs, having ~100 atoms, this will result in a huge number of vibrations that have to be accounted for. Typically, though, the vibrational features of a NFAs are approximated using one (or more) representative vibrational mode. The vibrational energy of such a mode generally ranges from 100 to 200 meV, arising from vinyl-stretching modes, carbon-carbon modes, or other molecular motions characteristic of the stiff backbone of the molecule. In the Franck-Condon (FC) approximation, where the transition dipole moment is considered a constant as a function of the nuclear coordinates, the absorption spectrum is given as:[3,4]

$$\alpha_{\mathrm{SM}}(E) = \alpha_{\mathrm{sat}} E \sum_m \rho_m \sum_n |\langle m|n\rangle|^2 \delta(E - (n-m)\hbar\omega - E_{\mathrm{SE}}). \quad \text{(S1)}$$

Here, ρ_m is the Boltzmann factor describing the population of vibrational mode m in the electronic ground state, α_{sat} is a merger of constants (including the transition dipole moment) describing the saturation value of the spectrum, δ is the Dirac function, and $|\langle m|n\rangle|^2$ is the FC factor:[5]

$$|\langle m|n\rangle|^2 = e^{-S} S^{(n-m)} \frac{m!}{n!} \left(\mathcal{L}_n^{(n-m)}(S)\right)^2,$$

which describes the wavefunction overlap between the vibrational ground and excited state. Here, $\mathcal{L}_k^{(\alpha)}(x)$ is the associated Laguerre polynomial and S is the HR factor described above. An exemplary absorption spectrum, calculated with Equation S1, is shown in Figure S2b. As already mentioned, the vibrational signature of an NFA is usually modeled with one representative high-frequency vibration.[6,7] Additional to this high-frequency motion, large molecules like NFAs exhibit significant low-frequency backbone vibrations, typically around 25 meV. These lower-energy modes are usually accompanied by large HR factors, which in turn produce a Gaussian-like lineshape (Figure S2c). This effect was originally described by Thomas Keil, who showed that low-frequency modes with high HR factors yield a FC spectrum resembling a Gaussian distribution, with mean energy and variation equal to:[5]

$$E_{\mathrm{C}} = E_{\mathrm{SE}} + \lambda_{\mathrm{l}}, \qquad \sigma_{\mathrm{d,Keil}}^2 = \lambda_{\mathrm{l}} \hbar\omega_{\mathrm{l}} \cdot \coth\left(\frac{\hbar\omega_{\mathrm{l}}}{2k_{\mathrm{B}}T}\right).$$

Here, $\hbar\omega_{\mathrm{l}}$ and λ_{l} represent the low-frequency vibrational energy and reorganization energy, respectively while E_{C} and $\sigma_{\mathrm{d,Keil}}$ denote the mean and standard deviation (or spectral linewidth) of the Gaussian distribution. The subscript "d" is used to emphasize that this linewidth is dynamic in nature due to its temperature dependence. Figure S2c shows the similarity between the absorption spectrum calculated with Equation S1 and the Gaussian approximation using Keil's approach. In the high-temperature limit ($\hbar\omega_{\mathrm{l}} \ll k_{\mathrm{B}}T$), the expression for σ_{d} simplifies to:

$$\sigma_{\mathrm{d,Marcus}}^2 = 2\lambda_{\mathrm{l}} k_{\mathrm{B}} T,$$

which is the same result obtained when describing the electronic transition within the framework of Marcus electron-transfer theory.[8,9] Now, using the Gaussian approximation for low-frequency vibrations, we can model the absorption spectrum of an NFA with two representative modes (one low- and one high-frequency) as:

$$\alpha_{\mathrm{SM}}(E) = \frac{\alpha_{\mathrm{sat}} E}{\sqrt{4\pi\sigma_{\mathrm{d}}^2}} \sum_m \rho_m \sum_n |\langle m|n\rangle|^2 \exp\left(-\frac{(E - (n-m)\hbar\omega - E_{\mathrm{SE}} - \lambda_{\mathrm{l}})^2}{2\sigma_{\mathrm{d}}^2}\right), \quad \text{(S2)}$$

where the Dirac function is replaced by the Gaussian originating from the low-frequency vibration.

The final step in constructing the model is to adapt it to describe the absorption spectrum of an NFA in the thin film setting. When cast into a film, the molecules will no longer be isolated and will experience a variation in conformation and environment, resulting in a distribution of singlet exciton gap energies. This distribution is assumed to be Gaussian in shape and is described by an excitonic density of states (DOS) with standard deviation σ_{s} and mean energy E_{gap}:

$$g_{\mathrm{DOS}}(E'_{\mathrm{SE}}) = \frac{N_{\mathrm{SE}}}{\sqrt{2\pi\sigma_{\mathrm{s}}^2}} \exp\left(-\frac{\left(E'_{\mathrm{SE}} - E_{\mathrm{gap}}\right)^2}{2\sigma_{\mathrm{s}}^2}\right).$$

Here, N_{SE} is the excitonic state number density, E'_{SE} is the singlet exciton energy of an individual molecule in the film. The standard deviation of this DOS (σ_{s}) is referred to as the static excitonic disorder. The term "static" points to the fact that variations in molecular conformation and

environment are essentially frozen solid at the time of deposition resulting in σ_{s} being temperature independent (within the investigated range). Now, to arrive at the absorption spectrum of the thin film, we will need to implement this gaussian distribution of the exciton energies into Equation S2. This can be achieved by performing a convolution of the single-molecule FC spectrum with the Gaussian DOS:

$$\alpha(E) = \int_0^\infty \alpha_{\mathrm{SM}}(E'_{\mathrm{SE}}, E) g_{\mathrm{DOS}}(E'_{\mathrm{SE}}) dE'_{\mathrm{SE}}\,.$$

In other words, the absorption spectrum of the thin film is obtained by integrating the absorption spectrum of the single molecule (which is a function of the exciton energy) over the distribution of exciton energies. This integral can be solved analytically, yielding the following expression for the absorption spectrum of a thin film with a representative high- and low-frequency vibrational mode:

$$\alpha(E) = \frac{\alpha_{\mathrm{sat}} E}{\sqrt{2\pi\left(\sigma_{\mathrm{s}}^2 + \sigma_{\mathrm{d}}^2\right)}} \sum_m \rho_m \sum_n |\langle m|n\rangle|^2 \exp\left(-\frac{\left(E - (n-m)\hbar\omega - E_{\mathrm{gap}} - \lambda_{\mathrm{l}}\right)^2}{2\left(\sigma_{\mathrm{s}}^2 + \sigma_{\mathrm{d}}^2\right)}\right). \quad \text{(S3)}$$

This expression is essentially just a sum of weighted Gaussians with mean energy $(n-m)\hbar\omega + E_{\mathrm{gap}} + \lambda_{\mathrm{l}}$ and variance $\sigma_{\mathrm{s}}^2 + \sigma_{\mathrm{d}}^2$. This allowed the implementation of the Sum-of-Gaussians (SoG) method, detailed in the main text. More precisely, when an experimental thin film absorption spectrum is fitted with the SoG procedure, the extracted variation σ^2 is equal to $\sigma_{\mathrm{s}}^2 + \sigma_{\mathrm{d}}^2$ (in the current framework). Since the analytical expression for σ_{d}^2 is known, temperature dependent measurements of the thin film absorption spectra allow for direct determination of σ_{s}, λ_{l}, and optionally ω_{l}, which is the goal of our study.

Finally, as detailed in the main text, an effective FC model can be constructed from the SoG fitting results. To calculate the effective FC absorption spectrum, Equation S3 can be used with parameters: $\sigma_{\mathrm{s}}^2 + \sigma_{\mathrm{d}}^2 = \sigma^2$, $E_{\mathrm{gap}} + \lambda_{\mathrm{l}} = E_0$, $\hbar\omega = \hbar\omega_{\mathrm{eff}} = |E_0 - E_1|$, and $S = S_{\mathrm{eff}} = I_1/I_0$. Here, σ, E_0, E_1, I_0, and I_1 are the parameters extracted using the SoG procedure.

SI 3. Room temperature absorption spectra and extracted optical parameters

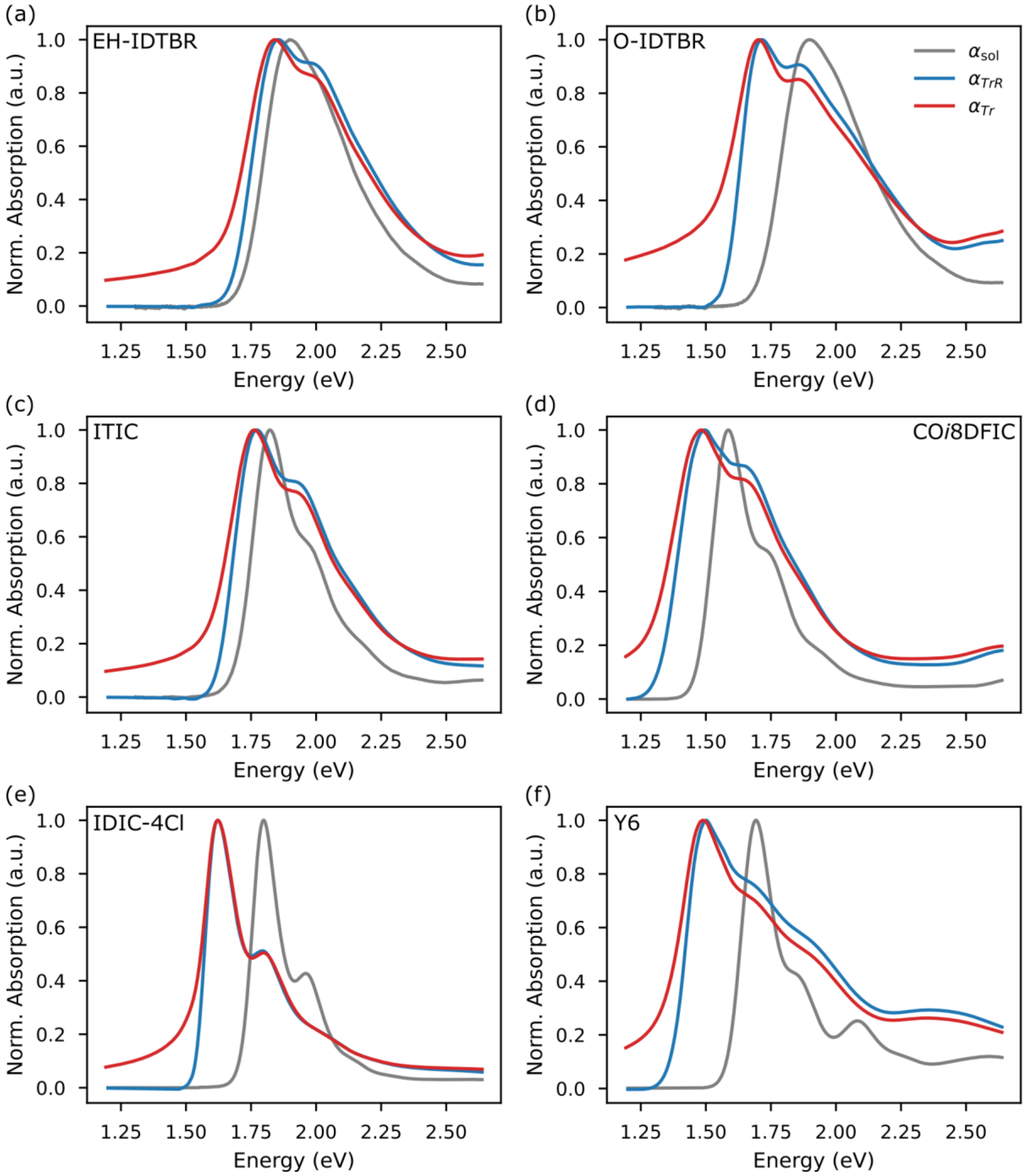

Figure S3. Normalized absorption spectra of a) EH-IDTBR, b) O-IDTBR, c) ITIC, d) CO*i*8DFIC, e) IDIC-4Cl, and f) Y6, in thin film setting (red and blue) and dilute chloroform solution (α_{sol}, gray). Thin film absorption spectra were obtained through the Beer-Lambert law considering only the transmittance (α_{Tr}, red) or both the transmittance and reflectance (α_{TrR}, blue). All spectra were determined at 293 K.

Table S1. First peak energies, effective FC (pseudo-)parameters, and linewidth for all investigated NFAs. The parameters were extracted from normalized absorption spectra, measured at 293 K, in thin film setting and dilute chloroform solution (α_{sol}) using the SoG procedure. For the thin film parameters, two different absorption spectra were characterized. Here, the absorption spectra were obtained through the Beer-Lambert law either considering only the transmittance (α_{Tr}) or both the transmittance and reflectance (α_{TrR}).

	Molecule	E_0 [eV]	$\hbar\omega_{\mathrm{eff}}$ [meV]	S_{eff}	σ [meV]
From α_{sol}	EH-IDTBR	1.875	0.163	0.669	79.86
	O-IDTBR	1.867	0.166	0.670	83.45
	ITIC	1.820	0.169	0.474	66.95
	CO*i*8DFIC	1.585	0.162	0.475	61.42
	IDIC-4Cl	1.803	0.154	0.411	49.37
	Y6	1.696	0.175	0.381	59.48
From α_{Tr}	EH-IDTBR	1.814	0.205	0.686	95.29
	O-IDTBR	1.684	0.207	0.688	89.93
	ITIC	1.746	0.206	0.618	88.57
	CO*i*8DFIC	1.456	0.214	0.642	94.99
	IDIC-4Cl	1.626	0.188	0.448	64.35
	Y6	1.482	0.220	0.557	91.39
From α_{TrR}	EH-IDTBR	1.833	0.179	0.783	79.74
	O-IDTBR	1.707	0.169	0.798	71.05
	ITIC	1.763	0.180	0.699	74.80
	CO*i*8DFIC	1.477	0.191	0.729	82.38
	IDIC-4Cl	1.634	0.169	0.494	55.05
	Y6	1.507	0.197	0.653	78.57

SI 4. Spectral data and temperature dependent fits for all investigated materials

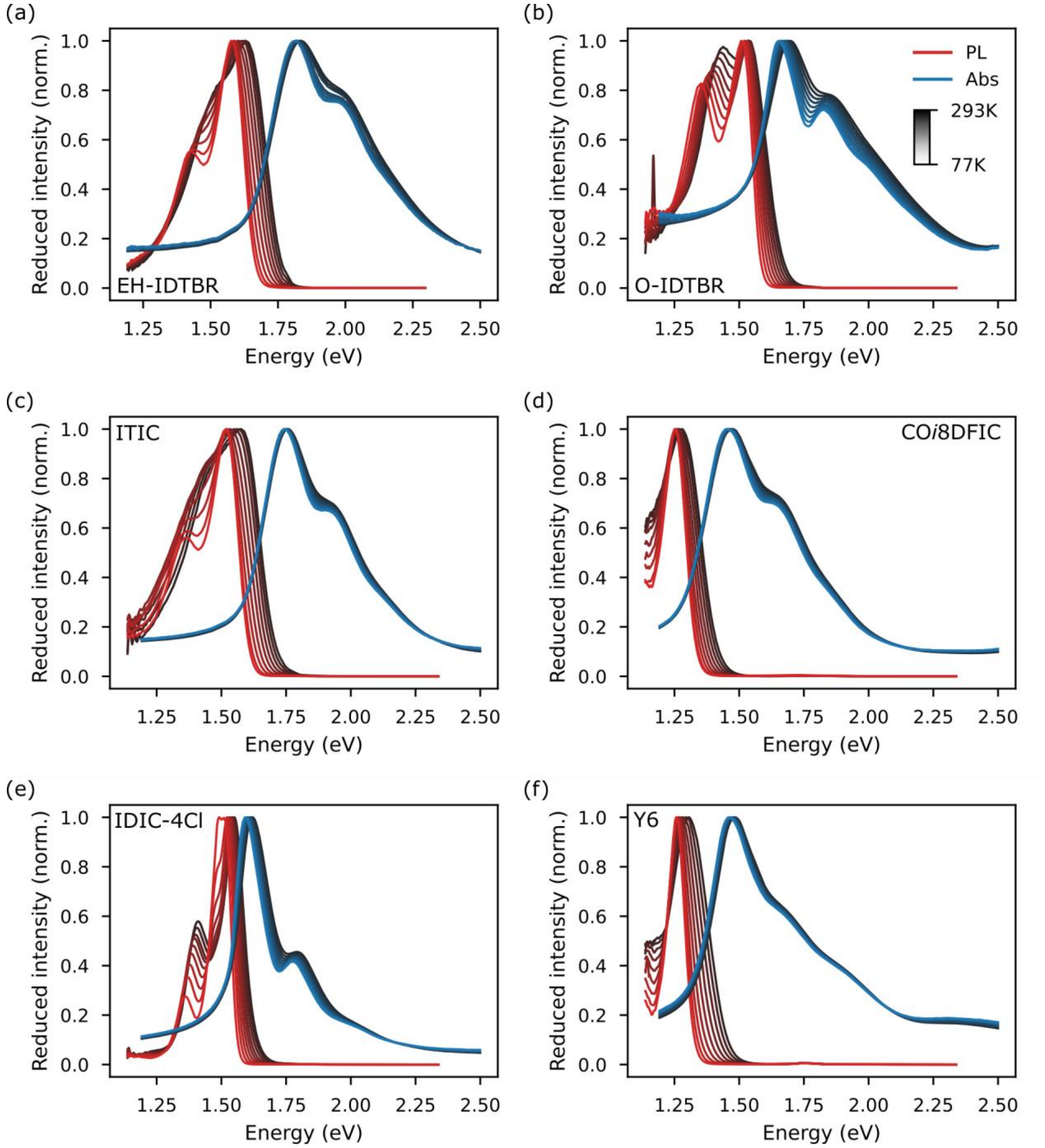

Figure S4. Reduced and normalized temperature dependent PL (red) and absorption (blue) spectra of a) EH-IDTBR, b) O-IDTBR, c) ITIC, d) CO*i*8DFIC, e) IDIC-4Cl, and f) Y6, in thin film setting. The temperature difference between two consecutive spectra is 27 K, going from 293 K to 77 K.

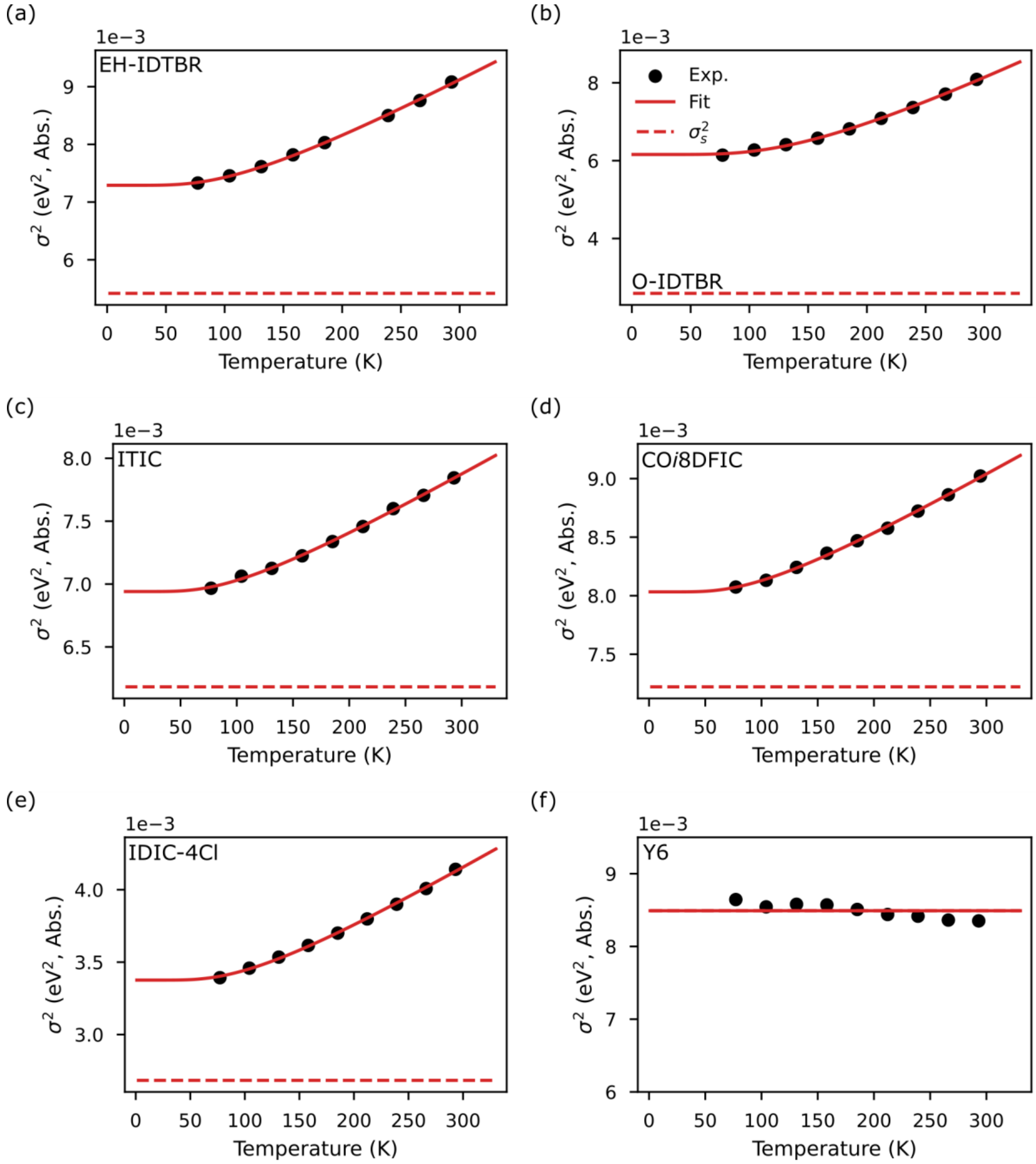

Figure S5. Square of the absorption linewidth as a function of temperature for a) EH-IDTBR, b) O-IDTBR, c) ITIC, d) CO*i*8DFIC, e) IDIC-4Cl, and f) Y6, in thin film setting, fitted with Equation (1) using Keil's expression for σ_{d}^2. The dashed line indicates the contribution from the static excitonic disorder.

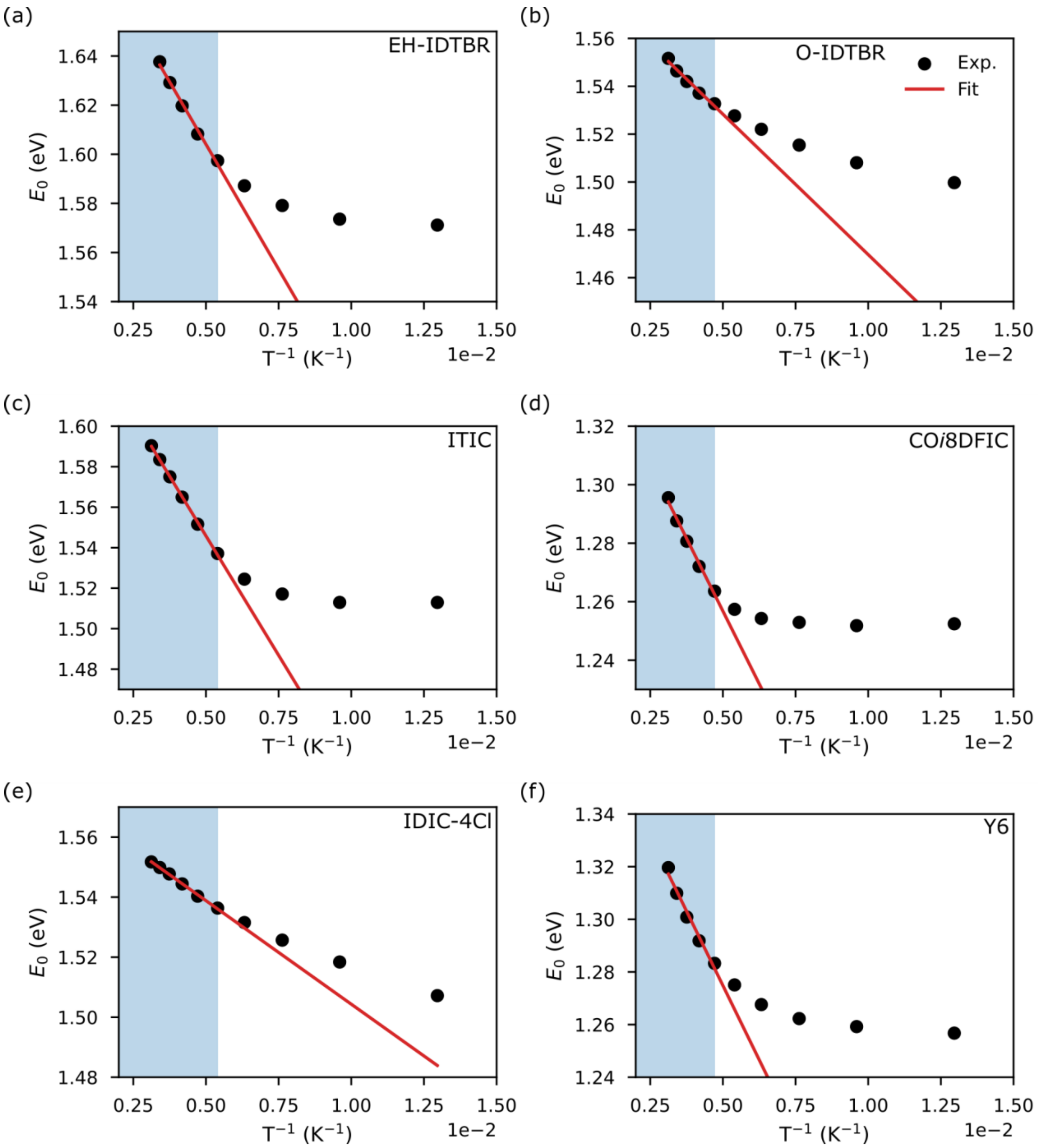

Figure S6. First peak energy of the PL spectrum as a function of inverse temperature $E_0(T^{-1})$ for a) EH-IDTBR, b) O-IDTBR, c) ITIC, d) CO*i*8DFIC, e) IDIC-4Cl, and f) Y6, in thin film setting, obtained from SoG fitting. The data is linearly fitted for σ_S (red line) where only PL data with temperatures above T_C (light blue area) is used for fitting. Here, T_C is determined as the point where $E_0(T^{-1})$ deviates from linearity.

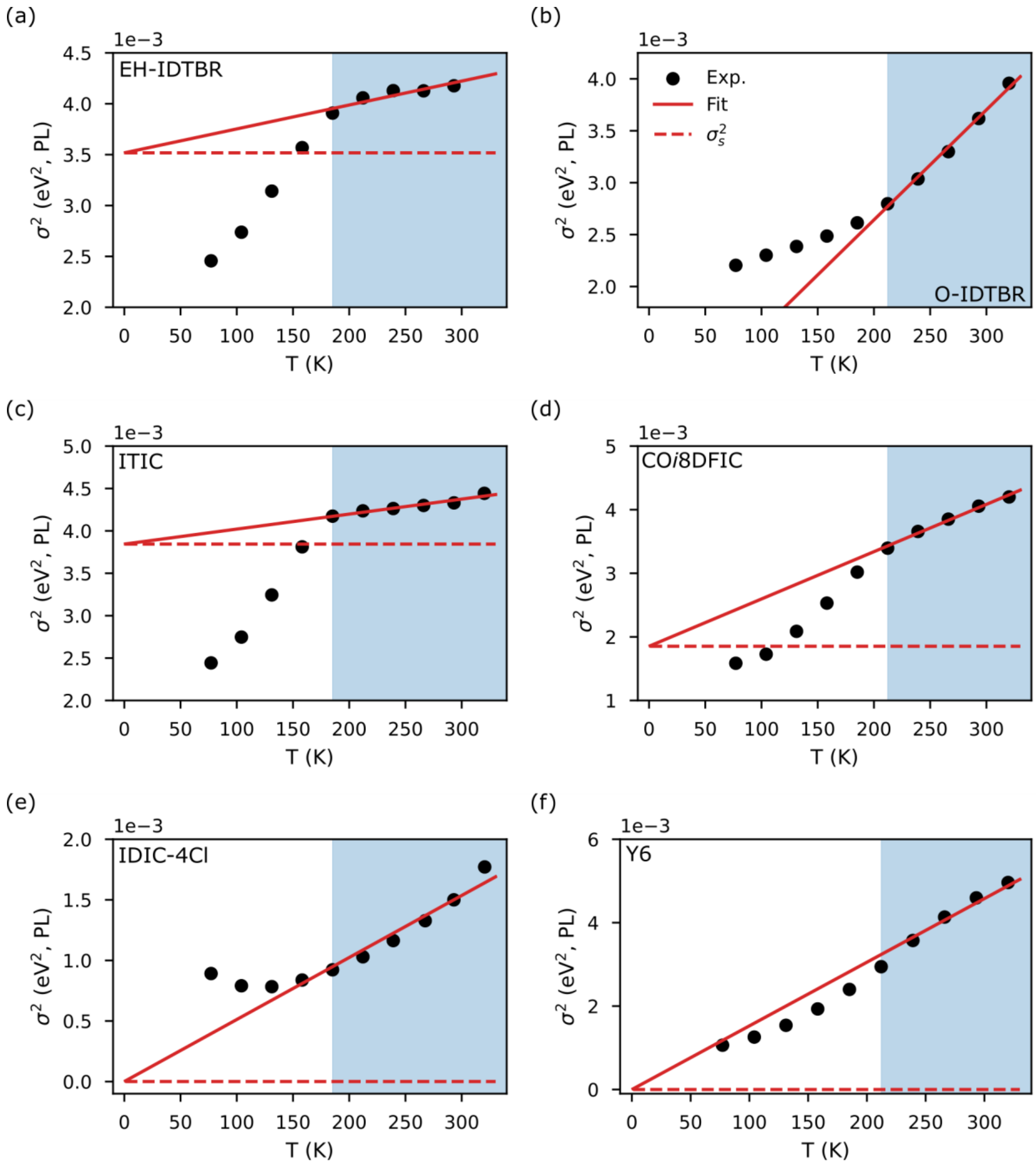

Figure S7. Square of the PL linewidth as a function of temperature for a) EH-IDTBR, b) O-IDTBR, c) ITIC, d) CO*i*8DFIC, e) IDIC-4Cl, and f) Y6, in thin film setting, fitted with Equation (1) using Marcus' expression for σ_{d}^2. The dashed line indicates the contribution from the static excitonic disorder. Only PL data with temperatures above T_{C} (light blue area) is used for fitting. Here, T_{C} is determined from the temperature dependence of the first peak energy E_0.

SI 5. Difference in absorption determined from transmittance and from both transmittance and reflectance

The temperature dependent thin film absorption spectra of the six investigated NFAs are determined by measurement of their transmittance spectra. This transmittance spectrum is then converted to an absorption spectrum using the Beer-Lambert law:

$$\alpha_{Tr} = -\frac{\ln(Tr)}{d},$$

where α represents the absorption spectrum, Tr the transmittance spectrum, and d the thickness of the thin film. Determination of the thickness is not necessary since only normalized spectra are used for analysis. The thickness of the film will induce interference effects though (see the following section for more details). A more accurate absorption spectrum is obtained when the reflectance spectrum R is also considered in the Beer-Lambert law:

$$\alpha_{TrR} = -\frac{\ln\left(\frac{Tr}{1-R}\right)}{d}.$$

A comparison between the absorption spectra obtained using only the transmittance (α_{Tr}), and the absorption spectra obtained using both transmittance and reflectance (α_{TrR}) is shown in Figure S3 for all investigated NFAs. Additionally, all spectra are fitted using the SoG procedure with the resulting parameters presented in Table S1.

When omitting the reflectance from the Beer-Lambert law, five main effects can be discerned from Figure S3 and the differences in optical parameters (Table S2): (i) The absorption edge significantly broadens and does not go to zero at low energies, (ii) the energy of the first absorption peak decreases 19 meV on average, (iii) the vibronic shoulder decreases in intensity resulting from an average 0.09 decrease of the effective HR factor, (iv) the effective vibrational energy increases 25 meV on average, and (v) the linewidth of the spectrum increases 14 meV on average. The impact of the effect (i) can largely be negated by choice of the right fitting range. As a rule of thumb, our fitting range for the SoG procedure starts at the half-maximum of the first peak in order to omit the artificially broad absorption onset. Effects (ii)-(iv) result in an underestimation of the effective HR factor and first peak energy, and an overestimation of the effective vibrational energy. It is important to keep in mind, here, that the effective FC parameters are pseudo-parameters and are only used for comparison of the spectral shapes. Finally, the effect (v) results in an overestimation of the linewidth of the spectrum. This will have the largest impact on our study as an overestimation in linewidth will result in an overestimation of the static excitonic disorder when extracted from $\sigma^2(T)$ fitting in absorption.

Table S2. Relative differences in extracted first peak energy, FC (pseudo-)parameters, and linewidth, for the six investigated NFAs determined from α_{Tr} and α_{TrR} measured at 293 K.

Molecule	$\Delta E_{0,Tr-TrR}$ [meV]	$\Delta\hbar\omega_{Tr-TrR}$ [meV]	ΔS_{Tr-TrR}	$\Delta\sigma_{Tr-TrR}$ [meV]
EH-IDTBR	-19.52	26.28	-0.10	15.56
O-IDTBR	-23.34	37.90	-0.11	18.88
ITIC	-17.21	25.95	-0.08	13.78
CO*i*8DFIC	-21.46	22.95	-0.09	12.60
IDIC-4Cl	-7.99	18.71	-0.05	9.30
Y6	-24.63	22.86	-0.10	12.82

SI 6. Thickness dependent interference effects in absorption

To investigate the influence of thin film interference on $\sigma^2(T)$ fitting in absorption, we simulated the transmittance and reflectance spectra of Y6 and ITIC as a function of film thickness. These simulations were performed using OpenFilters, an open-source software package that calculates transmittance and reflectance based on the refractive index (n) and extinction coefficient (k) of the materials.[10] Consequently, determining the n and k values for Y6 and ITIC was a prerequisite for this analysis.

For Y6, we employed the methodology developed by Kerremans et al., which utilizes the transmittance (and optionally the reflectance) of two samples with different, known thicknesses to extract the complex refractive index (*i.e.* n and k).[11] This iterative process adjusts n and k values to ensure the simulated transmittance spectra, derived from transfer matrix modeling, optimally reproduces the experimental transmittance spectra provided as input. To facilitate this process, we fabricated two Y6 thin film samples using chloroform solutions of 5 mg/mL and 10 mg/mL, resulting in films with thicknesses of 37 nm and 67 nm, respectively, as measured by profilometry (Bruker DekTakXT). Using the transmittance and reflectance spectra of these samples, we determined the n and k values with the Kerremans et al. software. For ITIC, the complex refractive index was digitized from the Supporting Information of the Kerremans et al. study using WebPlotDigitizer.[11,12]

Now, making use of the complex indices of refraction, the transmittance and reflectance spectra of Y6 and ITIC were calculated. Here, the thin film was modeled as a borosilicate glass substrate (1 mm) with, on top, a 25 to 100 nm thick layer of the active materials. Subsequently, the absorption spectra of both materials, as a function of film thickness, were determined using the Beer-Lambert law. This was done in two ways: using only the transmittance to obtain α_{Tr}, and using both the transmittance and reflectance to obtain α_{TrR}. The absorption spectrum was also directly determined from the extinction coefficient as:[11]

$$\alpha_k(\lambda) = \frac{4\pi k(\lambda)}{\lambda},$$

which can be considered as the "true" absorption spectrum of the material, devoid of any interference effects. Figure S8 shows the simulated absorption spectra α_{Tr}, α_{TrR} and α_k for Y6 and ITIC as a function of film thickness. Here, the film thickness is varied from 25 to 100 nm in 5 nm increments as we expect, from experience, that the investigated thin film samples from the main text (spin cast from 10 mg/mL chloroform solution at 2000 RPM) will have thicknesses laying in this range. Subsequently, a SoG fit was performed on all spectra, the extracted linewidths as a function of film thickness are presented in Figure S9.

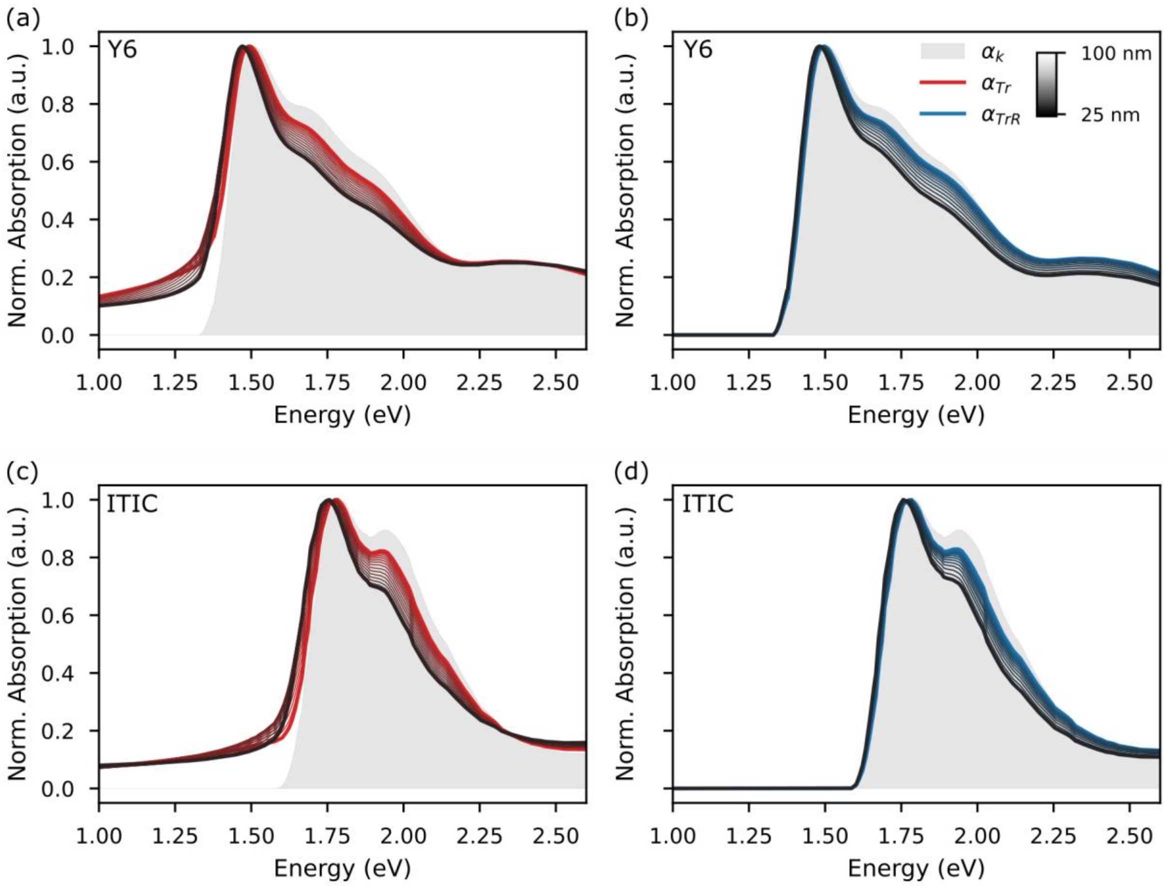

Figure S8. Simulated thin film absorption spectra for Y6 (a, b) and ITIC (c, d) for thicknesses ranging from 25 nm to 100 nm in steps of 5 nm. Absorption spectra were obtained through the Beer-Lambert law taking either the transmittance (red) or both the transmittance and reflectance (blue) into account. The absorption spectrum α_k devoid of interference effects is displayed in gray for comparison.

From this analysis, we can conclude that the linewidths extracted from α_{Tr}, when compared to the extracted linewidths from α_{TrR}, generally experience a larger influence from the thickness dependent interference effect, fluctuating 6-8 meV across the investigated thickness range. Moreover, extracted linewidths from α_{TrR} spectra are generally much closer to the true linewidth, deviating no more than 3 meV across the investigated thickness range. Linewidth extracted from α_{Tr}, on the other hand, experience a much larger deviation of 3-10 meV. This observation does not come unexpected as in the previous section we already concluded that the linewidth extracted from α_{Tr} is on average 14 meV larger than the linewidth extracted from α_{TrR} (Table S2). As a result, we can conclude that the error induced by thickness dependent interference effects is largely overshadowed by the error induced by omittance of the reflectance in calculation of the absorption spectrum through the Beer-Lambert law. The conclusion from the previous section thus remains valid: the total linewidth in the $\sigma^2(T)$ fitting procedure for absorption is overestimated, resulting in an overestimation of the extracted static excitonic disorder.

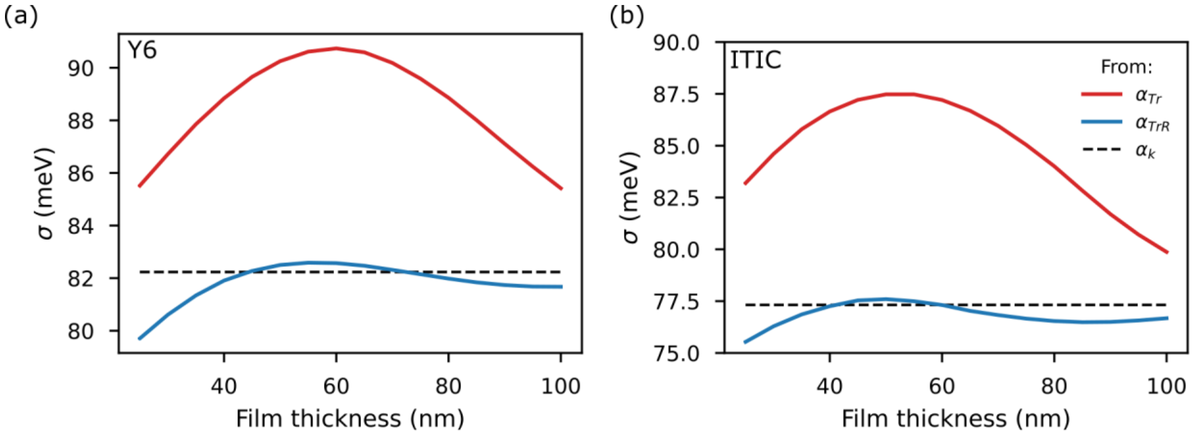

Figure S9. Total linewidth of the simulated α_{Tr} (red), α_{TrR} (blue) and α_k (dashed black) spectra of Y6 (a) and ITIC (b) as a function of film thickness. The linewidths were extracted using the SoG fitting procedure.

SI 7. Impact of self-absorption and cavity effects in PL

To estimate the impact of self-absorption and cavity effects on the extracted static excitonic disorder from $E_0(T^{-1})$ fitting in PL, we will draw upon the findings of van der Pol et al.[13] Their study examined how these effects influence the outcoupled peak maxima of electroluminescence (EL) spectra in OSCs. We will apply their results to the PL spectra of neat NFA thin films expecting them to be transferable. Additionally, the cavity effect in PL is expected to be less pronounced than in EL, as the active layer in OSCs is typically enclosed by multiple additional layers, often including a reflective top electrode.

In the method proposed by van der Pol et al. they start with defining the optical properties of a model active material. Here, the extinction coefficient (k) is taken to be a gaussian with its maximum at energy E_C. The refractive index (n) is then determined using the Kramers-Kronig transformation. Finally, the intrinsic emission spectrum (Em_in) is determined using a transformation loosely based on the reciprocity theorem. Using the optical environment, determined by the surrounding layers, the outcoupled emission spectrum is calculated (Em_out). Now, the difference in peak energy ΔE_em between the intrinsic and outcoupled emission spectrum is determined:

$$\Delta E_\mathrm{em} = E_\mathrm{in} - E_\mathrm{out},$$

where E_in and E_out are the maximum peak energies of the intrinsic and outcoupled emission, respectively. Now, van der Pol et al. determined that ΔE_em is influenced by three mains factors: active layer thickness, peak energy of the extinction coefficient E_C, and the emission profile/origin in the active layer. Using these three factors, van der Pol et al. calculated four heatmaps with ΔE_em as a function of active layer thickness and E_C. For each heatmap, a different emission profile was chosen with gaussian emission either originating from the front, middle, or back of the active layer. The fourth heatmap was calculated with an emission profile which was constant across the active layer. For PL, with laser excitation at the front of the sample, the emission profile is expected to be exponential, decaying from the front to the back of the active layer surface. For this reason, we will take the results of van der Pol et al. calculated for the front surface as a basis of the following analysis.

For determination of the static excitonic disorder from PL measurements, the PL spectrum is first measured as a function of temperature. Then, each spectrum is fitted using the SoG method from which the first peak energy E_0 as a function of inverse temperature T^{-1} is used to determine the static excitonic disorder σ_s and the mean gap energy E_gap. We can now use the results of van der Pol et al. to estimate the induced error on σ_s and E_gap due to self-absorption and cavity effects. Based on the heatmaps from van der Pol et al. we (generously) estimate the outcoupled peak energy to be redshifted by 50 meV for active layer thicknesses between 50 and 100 nm. Thus, to correct for self-

absorption and cavity effects we can simply add 50 meV to $E_0(T^{-1})$ as a correction factor before applying the fitting procedure. Furthermore, this will only result in a 50 meV increase of the extracted mean gap energy E_{gap} with no change in σ_{s} as it is determined from the slope of $E_0(T^{-1})$ (which is not influenced by a constant factor). As already mentioned, ΔE_{em} is not only influenced by thickness, but also by E_{C} and consequently E_{in}. Since E_{in} varies in our measurements due to the temperature variation, ΔE_{em} as a result also varies. Consequently, correcting for self-absorption and cavity effects by adding a constant correction energy of 50 meV might be too simplistic. Experimentally, E_0 only varies 50-100 meV throughout a temperature dependent measurement and through this range we can approximate the effect of ΔE_{em} variation to be negligible. As a result, we expect the impact of self-absorption and cavity effects to be negligible for the static excitonic disorder and translate in an approximate 50 meV underestimation of the mean gap energy E_{gap}.

References

1 E. B. Wilson, J. C. Decius and P. C. Cross, *Molecular Vibrations: The Theory of Infrared and Raman Vibrational Spectra*, Dover Publications, New York, Dover., 1980.

2 P. Atkins and R. Friedman, *Molecular Quantum Mechanics*, Oxford University Press, Oxford, 5th edn., 2011.

3 F. Santoro, A. Lami, R. Improta and V. Barone, *J Chem Phys*, 2007, 126, 184102.

4 J. Bloino, M. Biczysko, F. Santoro and V. Barone, *J Chem Theory Comput*, 2010, 6, 1256–1274.

5 T. H. Keil, *Physical Review*, 1965, 140, A601–A617.

6 S. Giannini, D. J. C. Sowood, J. Cerdá, S. Frederix, J. Grüne, G. Londi, T. Marsh, P. Ghosh, I. Duchemin, N. C. Greenham, K. Vandewal, G. D'Avino, A. J. Gillett and D. Beljonne, *Materials Today*, 2024, 80, 308–326.

7 N. J. Hestand and F. C. Spano, *Chem Rev*, 2018, 118, 7069–7163.

8 R. A. Marcus, *J Chem Phys*, 1956, 24, 966–978.

9 I. R. Gould, D. Noukakis, L. Gomez-Jahn, R. H. Young, J. L. Goodman and S. Farid, *Chem Phys*, 1993, 176, 439–456.

10 S. Larouche and L. Martinu, *Appl Opt*, 2008, 47, C219.

11 R. Kerremans, C. Kaiser, W. Li, N. Zarrabi, P. Meredith and A. Armin, *Adv Opt Mater*, 2020, 8, 2000319.

12 Ankit Rohatgi, WebPlotDigitizer 4.8, https://apps.automeris.io/wpd4/, (accessed 3 February 2025).

13 T. P. A. van der Pol, K. Datta, M. M. Wienk and R. A. J. Janssen, *Solar RRL*, 2022, 6, 2200872.
